# Predictive control of selective secondary alcohol oxidation of glycerol on NiOOH

**DOI:** 10.1038/s41467-022-33637-7

**Published:** 2022-10-04

**Authors:** McKenna K. Goetz, Michael T. Bender, Kyoung-Shin Choi

**Affiliations:** Department of Chemistry, University of Wisconsin-Madison, Madison, WI 53706 USA

**Keywords:** Electrocatalysis, Electrocatalysis, Catalytic mechanisms

## Abstract

Many biomass intermediates are polyols and selectively oxidizing only a primary or secondary alcohol group is beneficial for the valorization of these intermediates. For example, production of 1,3-dihydroxyacetone, a highly valuable oxidation product of glycerol, requires selective secondary alcohol oxidation. However, selective secondary alcohol oxidation is challenging due to its steric disadvantage. This study demonstrates that NiOOH, which oxidizes alcohols via two dehydrogenation mechanisms, hydrogen atom transfer and hydride transfer, can convert glycerol to 1,3-dihydroxyacetone with high selectivity when the conditions are controlled to promote hydrogen atom transfer, favoring secondary alcohol oxidation. This rational production of 1,3-dihydroxyacetone achieved by selectively enabling one desired dehydrogenation pathway, without requiring alteration of catalyst composition, demonstrates how comprehensive mechanistic understanding can enable predictive control over selectivity.

## Abstract (web summary)

While valuable chemicals can be obtained from biomass electro-oxidation, the mechanisms can be challenging to discern. Here, authors show that explicit control of electrochemical dehydrogenation mechanisms enables selective secondary alcohol oxidation of glycerol to produce 1,3-dihydroxyacetone.

## Introduction

**Fig. 1:**
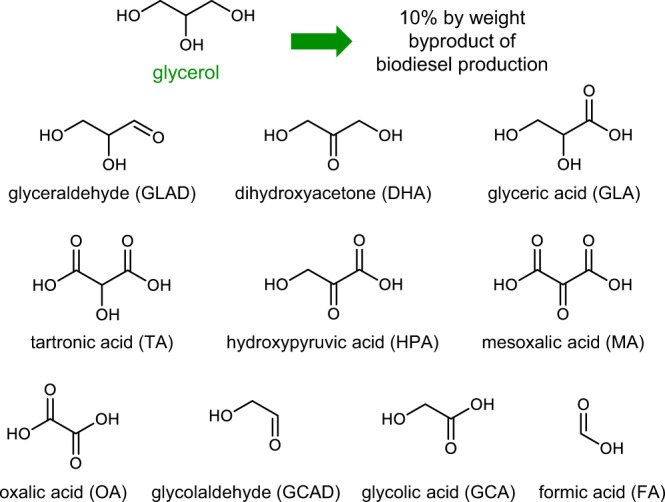
Selected products from glycerol oxidation. C1–C3 molecules that can be obtained from oxidative valorization of glycerol.

Biomass is an attractive source of environmentally friendly fuels and building block chemicals^1–3^. For example, biodiesel, which is produced via the transesterification of biomass-derived triglycerides, has been identified as a renewable, biodegradable, and non-toxic replacement for traditional diesel oil^4^. However, biodiesel production co-generates glycerol as a 10% by weight byproduct, far outweighing the current demand for glycerol and consequently limiting the price of biodiesel. The utility of the glycerol byproduct can be significantly increased through oxidative upgrading as nearly every product of glycerol oxidation is more valuable than glycerol itself (Fig. 1)^5^. The difficulty with this approach is in the efficient and selective oxidation of glycerol to a desired product. Structurally, glycerol contains three alcohol functional groups—two primary and one secondary—and selectively oxidizing one of these very similar functional groups while preventing further oxidation can be challenging. The oxidation of glycerol to 1,3-dihydroxyacetone (DHA) via selective oxidation of its secondary alcohol group is particularly interesting because DHA has been identified as one of the most valuable targets^6,7^. However, selective secondary alcohol oxidation is much more difficult than selective primary alcohol oxidation due to increased steric hinderance. Considering that many other biomass intermediates are polyols that possess both primary and secondary alcohol groups, developing methods to achieve selective secondary alcohol oxidation can have broad applications for biomass upgrading.

Electrochemical glycerol oxidation has recently received a great deal of attention as it can be coupled to renewable sources of electricity and employ water as the reaction medium^8^. The majority of previous studies of electrochemical glycerol oxidation have used precious metal (e.g., Pt, Pd, Ag, or Au)-based catalysts^5,6,9,10^, of which only a few have selectively produced DHA (e.g., Bi or Sb modified Pt catalysts)^11–16^. As for non-noble metal-containing electrocatalysts, Ni-based ones have been the most extensively investigated^17–30^. The active catalyst layer for most of these Ni-based electrocatalysts is NiOOH, which is known for its ability to oxidize primary alcohols to the corresponding carboxylic acids^31–33^. Almost all of the studies using Ni-based electrocatalysts for glycerol oxidation have so far been carried out under highly basic conditions (pH ≥ 13) and report formic acid (FA) as the major product^26,34^, suggesting that glycerol oxidation on NiOOH involves significant C–C cleavage reactions and therefore cannot be used to selectively generate C3 or C2 oxidation products. However, we note that in highly basic media some C3 and C2 oxidation products such as glyceraldehyde (GLAD), DHA, and glycolaldehyde (GCAD) are not chemically stable and will decompose to produce FA and other products even without the presence of a catalyst (Supplementary Fig. 1)^35,36^. Thus, investigation of glycerol oxidation on NiOOH at lower pH where all products are stable is needed to more accurately investigate the ability of NiOOH to produce C3 and C2 products.

**Fig. 2:**
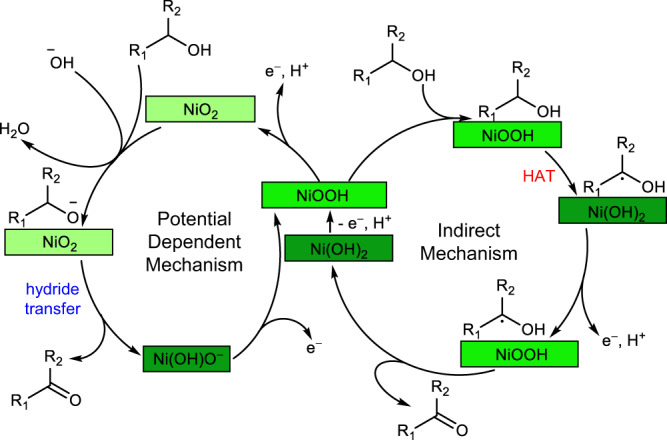
Simplified representations of two possible alcohol dehydrogenation mechanisms on NiOOH electrodes. R_1_, R_2_ = aliphatic carbon chain, aromatic carbon chain, or H. HAT = hydrogen atom transfer. For 2° alcohols, oxidation stops at the ketone product. For 1° alcohols, oxidation may proceed to the carboxylic acid via analogous oxidation of the hydrated geminal diol form of the initially formed aldehyde.

In addition to ensuring product stability, there is an even more compelling reason to study glycerol oxidation on NiOOH at lower pH. The oxidation of an alcohol to an aldehyde or ketone involves the removal of two H–atoms, one from the α-C and the other from the O of the hydroxy group. Recently, we revealed that NiOOH has two electrochemical dehydrogenation mechanisms for oxidizing alcohols (Fig. 2) whose rates display unique pH and potential dependencies^37,38^. The first is the indirect mechanism that has been known for 50 years^31,39^. In this mechanism, Ni^3+^ sites in electrochemically generated NiOOH are known to be the active centers that chemically oxidize organic species regardless of any applied potential. The rate determining step of this mechanism is reported to involve the transfer of an H–atom from the α-C to NiOOH via hydrogen atom transfer (HAT)^31,39^, resulting in formation of an α-C radical and conversion of NiOOH to Ni(OH)_2_. This is assumed to be followed by rapid removal of the H–atom from the O–H by an additional NiOOH site to produce the aldehyde or ketone. For this indirect mechanism, the applied potential only regenerates the chemical oxidant NiOOH from Ni(OH)_2_ and doesn’t directly oxidize the alcohol (thus the name indirect oxidation). The second alcohol dehydrogenation mechanism is the potential dependent (PD) mechanism that was revealed by our group in 2020^37,38^. This mechanism is enabled when Ni^4+^ is present and therefore is promoted in a more positive potential region. Dehydrogenation in the PD mechanism is proposed to occur through a net hydride transfer (1 H^+^, 2 e^–^) from the α-C of an alcohol group to Ni^4+^ and a proton transfer from the O–H to hydroxide in solution. The proton transfer can occur either before or concurrently with the hydride transfer. This mechanism requires an applied potential to drive the hydride transfer reaction whose rate is potential-dependent (thus the name PD oxidation).

Using our understanding of the two different dehydrogenation mechanisms that can occur on NiOOH, we can rationally formulate oxidation conditions to control the selectivity for primary and secondary alcohol oxidation. Our previous studies showed that aliphatic primary alcohols (ethanol and 1-butanol) are oxidized mainly through PD oxidation^38^, meaning dehydrogenation of primary alcohols via the indirect mechanism is much slower. This is likely because the homolytic cleavage of the strong C_α_–H bond of a primary alcohol required for HAT is difficult and the resulting C_α_ radical is unstable. We postulate that in comparison, secondary alcohols should be easier to oxidize via the indirect mechanism since the C_α_–H bond is weaker and the resultant radical is relatively more stable^40^. This implies that if we provide oxidation conditions that suppress the PD mechanism relative to the indirect mechanism, we can offer a rational strategy to promote secondary alcohol oxidation over primary alcohol oxidation and convert glycerol to DHA on NiOOH.

Here, we demonstrate how pH and potential can be regulated to rationally control the dehydrogenation mechanisms on NiOOH to promote oxidation of the secondary alcohol of glycerol. We additionally discuss how pH and potential affect C–C cleavage during glycerol electrooxidation, as the current understanding of C–C cleavage reactions is sparse despite its criticality for controlling the selectivity toward DHA and other C1–C3 oxidation products. Our comprehensive investigation of a complicated set of electrochemical glycerol oxidation reactions enables the formulation of reaction conditions to maximize DHA production on the non-precious metal catalyst NiOOH with high selectivity and Faradaic efficiency.

## Results and discussion

### Analysis of linear sweep voltammograms

**Fig. 3:**
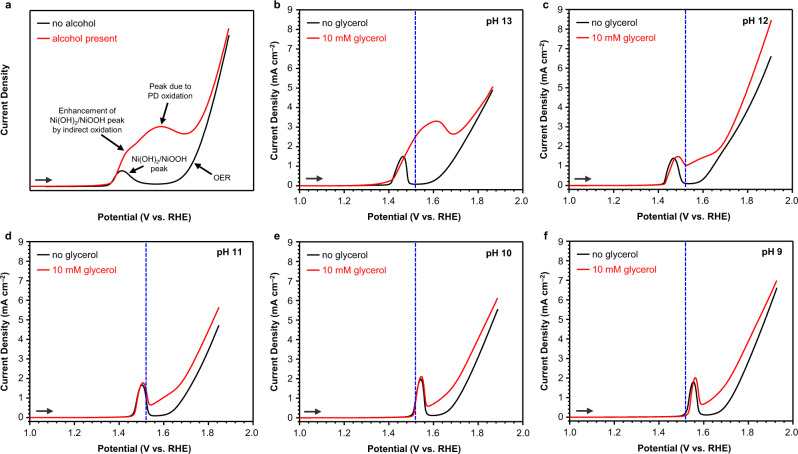
Linear sweep voltammograms (LSVs) of α-Ni(OH)_2_ films. **a** Schematic LSVs with (red) or without (black) an alcohol demonstrating expected anodic features. **b–f** LSVs obtained with (red) or without (black) 10 mM glycerol at pH 9-13 conditions. The blue dashed lines indicate 1.52 V_RHE_, one of the potentials used for constant potential electrolysis experiments, demonstrating how pH affects the Ni(OH)_2_/NiOOH peak position relative to a given constant potential.

PD oxidation requires proton transfer coupled with hydride transfer^37,38^. Therefore, its rate for glycerol oxidation is expected to decrease as pH decreases. On the other hand, indirect oxidation involves HAT and has no obvious reason to be pH dependent as long as the solution is basic enough to keep the regeneration of NiOOH from Ni(OH)_2_ (Ni(OH)_2_ + OH^–^ → NiOOH + H_2_O + e^–^) faster than the rate-limiting HAT step^37,38^. Therefore, the relative contribution from indirect glycerol oxidation current to the total glycerol oxidation current is expected to increase as pH decreases. We first qualitatively examined this postulation by obtaining linear sweep voltammograms (LSVs) in pH 9-13 solutions containing 10 mM glycerol (Fig. 3). The LSVs were recorded using amorphous α-Ni(OH)_2_ as the working electrode (WE) by sweeping the potential from the open circuit potential in the positive direction (α-Ni(OH)_2_ is the as-prepared precursor of NiOOH used in this study). A borate buffer was chosen for pH 9-12 solutions with K_2_SO_4_ as a supporting electrolyte to ensure consistency of anion types across a wide range of pH conditions (see Methods and Supplementary Fig. 2 for details). For pH 13, a pure 0.1 M KOH solution was used.

Figure 3a shows schematically how indirect and PD oxidation of an alcohol would appear in an LSV. When an LSV of α-Ni(OH)_2_ is recorded without an alcohol present, there are two features: an oxidation peak for the conversion of Ni(OH)_2_ to NiOOH and an oxidation wave for the oxygen evolution reaction (OER). When an alcohol is present, indirect oxidation of the alcohol appears as an enhancement of the Ni(OH)_2_/NiOOH oxidation peak. This is because NiOOH generated during an LSV immediately serves as a chemical oxidant to oxidize the alcohol via the indirect mechanism and is itself reduced back to Ni(OH)_2_ in the process. As such, more current is needed to regenerate NiOOH during the LSV, resulting in current enhancement of the Ni(OH)_2_/NiOOH oxidation peak. Since the anodic current for indirect oxidation is only due to regeneration of NiOOH from Ni(OH)_2_ and not direct alcohol oxidation, the degree of enhancement of the Ni(OH)_2_/NiOOH peak is directly proportional to the rate of indirect oxidation of the given alcohol. On the other hand, PD oxidation of alcohols, which requires the formation of Ni^4+^ and is potential-dependent, is most evident as a current enhancement in a wider potential range between the Ni(OH)_2_/NiOOH peak and the onset of OER.

The LSV obtained with glycerol in pH 13 (Fig. 3b) shows considerable current enhancement due to PD oxidation between the Ni(OH)_2_/NiOOH peak and the oxidation wave for OER. This current feature merges with the Ni(OH)_2_/NiOOH peak, making it difficult to examine any enhancement of the Ni(OH)_2_/NiOOH peak caused by indirect oxidation of glycerol at this pH. When the pH is lowered to 12 (Fig. 3c), the current for PD oxidation decreases drastically, revealing the Ni(OH)_2_/NiOOH peak, which is only slightly enhanced when glycerol is present. This enhancement, though, is still greater than those observed with primary aliphatic alcohols such as ethanol and 1-butanol that are oxidized mainly through PD oxidation^37,38^. We note that the Ni(OH)_2_/NiOOH peak is shifted slightly in the positive direction in the presence of glycerol because glycerol adsorbs on Ni(OH)_2_, affecting the oxidation potential Ni(OH)_2_. This phenomenon has been observed with other organic species that adsorb strongly on Ni(OH)_2_^41^. Nonetheless, in all cases, oxidation of Ni(OH)_2_ is required before glycerol oxidation can occur (Supplementary Fig. 3)^31^.

In pH 9-11 solutions (Fig. 3d–f), the current due to PD oxidation is further suppressed while the enhancement of the Ni(OH)_2_/NiOOH peak by indirect oxidation is more or less pH-independent, implying that it may be possible to increase the relative contribution of indirect oxidation to the total current by lowering the pH, as predicted. More quantitative analyses of the contributions from indirect and PD oxidation in these pH regions require an electrochemical rate deconvolution method discussed below.

The LSV results also revealed that in addition to suppressing the deprotonation of the alcohol group needed for PD oxidation, lowering the pH also suppresses PD oxidation by altering the redox potential for the Ni(OH)_2_/NiOOH couple. If the oxidation of Ni(OH)_2_ to NiOOH displayed Nernstian behavior (i.e., the redox peak shifted by –59 mV/pH when pH increased by one unit), the peak would always appear at the same potential vs. the reversible hydrogen electrode (RHE) as the RHE scale already compensates for the Nernst shift. However, as shown in Fig. 3b–f, the Ni(OH)_2_/NiOOH peak shift is non-Nernstian and instead gradually shifts to the positive direction on the RHE scale as pH decreases. Thus, if glycerol oxidation is performed at a constant potential vs. RHE (V_RHE_), such as 1.52 V_RHE_ (shown as the blue dashed lines in Fig. 3b–f for each LSV), the applied potential relative to the Ni(OH)_2_/NiOOH peak becomes less positive as pH decreases. This makes PD oxidation (which requires Ni^4+^) less favorable at any fixed potential vs. RHE.

### pH effects on product distribution and mechanism

**Fig. 4:**
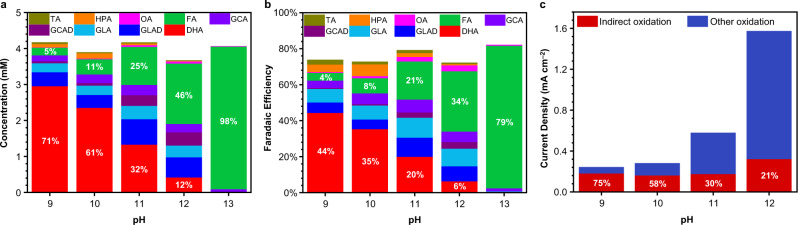
Glycerol oxidation results after electrolysis at a constant potential of 1.52 V_RHE_ under various pH conditions. **a** Product distributions showing concentrations after passing 18 C (the stoichiometric charge needed to convert ~25% glycerol to DHA). The percentages in white indicate the relative selectivities for DHA (red) and FA (green). **b** Faradaic efficiencies (FEs) after passing 18 C. The percentages in white indicate the FEs for DHA (red) and FA (green). **c** Current density from *I*_indirect_ (red, percentage shown in white) and *I*_other_ (blue) oxidation of glycerol under various pH conditions. TA = tartronic acid, gold, HPA = hydroxypyruvic acid, orange, OA = oxalic acid, pink; FA = formic acid, green, GCA = glycolic acid, bright purple, GCAD = glycolaldehyde, dark purple, GLA = glyceric acid, light blue, GLAD = glyceraldehyde, dark blue, DHA = 1,3-dihydroxyacetone, red.

To confirm various postulations that we constructed from the LSV results, we next performed constant potential glycerol oxidation at 1.52 V_RHE_ in 14 mL of pH 9-13 solutions containing 25 mM glycerol and quantified the products using high-performance liquid chromatography (HPLC). The results obtained after passing the same amount of charge (18 C) for each condition are shown in Fig. 4. Results obtained at other points throughout the electrolysis for each condition studied are compiled in Supplementary Figs. 4–8. In this study, we discuss the relative product selectivities using Eq. (1) instead of absolute selectivities (Eq. (6)) since we were unable to accurately quantify glycerol consumption due to poor separation by HPLC (see Methods). We believe that reporting these selectivities along with the absolute amounts and Faradaic efficiencies (FEs) for all products detected (Fig. 4a, b) is the best practice in reporting complex glycerol oxidation results involving C–C cleavage to form various C1–C3 products when the consumption of glycerol cannot be quantified.

1 $${{{{{\rm{Relative}}}}}}\; {{{{{\rm{Selectivity}}}}}}\,(\%)\,=\, \frac{{{{{{\rm{mol}}}}}}\; {{{{{\rm{of}}}}}}\; {{{{{\rm{specific}}}}}}\; {{{{{\rm{product}}}}}}}{{{{{{\rm{mol}}}}}}\; {{{{{\rm{of}}}}}}\; {{{{{\rm{all}}}}}}\; {{{{{\rm{products}}}}}}\; {{{{{\rm{detected}}}}}}}\, \times 100\%$$

The result obtained at pH 13 shows FA as the major product (Fig. 4a), which is consistent with previous reports^26,34^, indicating that C–C cleavage reactions are promoted in this highly basic solution. We additionally detected small amounts of glyceric acid (GLA) and glycolic acid (GCA), but no other products were detectable. However, as a few products such as DHA, GLAD and GCAD are chemically unstable at pH 13 (Supplementary Fig. 1) and some C3 and C2 products may have been further oxidized to FA, no detection does not necessarily mean no formation.

When the pH is lowered, a remarkable change is observed in the product selectivity. At pH 12, the production of FA is diminished considerably and other C3 and C2 products are now observed, suggesting that C–C cleavage reactions are less favored at pH 12 (Fig. 4a). Furthermore, DHA is observed as a product. However, the sum of other C3 oxidation products (GLA, GLAD, and tartronic acid (TA)) formed via primary alcohol oxidation is greater than the amount of DHA produced, indicating primary alcohol oxidation prevails over secondary alcohol oxidation at pH 12. We note that in addition to a pH difference, our pH 12 solution contains borate while our pH 13 solution does not. Previously, it has been reported that the presence of borate, which is known to form coordination complexes with polyols^42^, can help suppress C–C cleavage during glycerol electrooxidation^30^. Thus, we additionally carried out electrolysis at pH 13 in the presence of the same amount of borate present at pH 12 (Supplementary Fig. 9). Our results show that the selectivity for FA is decreased modestly (from 98 to 82%) but is still almost twice that observed at pH 12, implying that pH is a major factor affecting C–C cleavage. This point becomes even more evident when comparing the results obtained in pH 9-12 solutions (discussed below) containing the same amount of borate.

As the pH decreases further, FA production keeps decreasing while DHA production keeps increasing. Eventually, at pH 9 and 10 DHA becomes the major product, meaning secondary alcohol oxidation prevails over other reactions. At pH 9, DHA constitutes >70% of the detectable glycerol oxidation products, which is remarkable. The FEs for FA and DHA production are shown in Fig. 4b, which demonstrate the direct effect of pH on FA and DHA production (See Supplementary Methods and Supplementary Table 1 for FE calculation details). The sum of the FEs for all products shown in Fig. 4b is around 80% under all pH conditions. As 1.52 V_RHE_ is not sufficient to induce OER and all quantifiable solution products were included in Fig. 4b, this discrepancy is due to further oxidation of FA (and other surface adsorbed intermediates) to CO_2_(g), which, in our basic solutions, is rapidly trapped as HCO_3_^–^/CO_3_^2–^ that is difficult to quantify and was not included as a product in Fig. 4b. To support this hypothesis, we confirmed that FA can be further oxidized to CO_2_(g) (trapped as HCO_3_^–^/CO_3_^2–^) on NiOOH under our electrolysis conditions, as assessed by consumption of FA without producing any other detectable products (Supplementary Fig. 10).

We next sought to determine the origins of the high selectivity for DHA at lower pH conditions. Our hypothesis was that conditions favoring the indirect mechanism of alcohol oxidation on NiOOH would favor secondary alcohol oxidation and produce DHA. Therefore, we quantitatively analyzed what fraction of the total oxidation current is from indirect oxidation at 1.52 V_RHE_ for each pH using our electrochemical rate deconvolution method, which we developed to study indirect and PD oxidation of alcohols, aldehydes, and amines on NiOOH (details are in the Methods, Supplementary Methods, and our previous studies^37,38,41,43^). In previous studies, the total oxidation current (*I*_total_) could be deconvoluted simply into the dehydrogenation current via indirect oxidation (*I*_indirect_) and dehydrogenation current via PD oxidation (*I*_PD_) because the reactants were converted to their dehydrogenation products (e.g., the corresponding carboxylic acids or nitriles) with ~100% FE without any C–C cleavage reactions or CO_2_ formation. However, the electrolysis results reported here show that *I*_total_ for glycerol oxidation must also include the current used for C–C cleavage reactions (*I*_c–c_) and the current used to produce CO_2_ (*I*_CO2_) (Eq. (2)).

2 $${I}_{{{{{{\rm{total}}}}}}}\,={I}_{{{{{{\rm{indirect}}}}}}}+{I}_{{{{{{\rm{PD}}}}}}}+{I}_{{{{{{\rm{c}}}}}}{{{{{\rm{\hbox{-}}}}}}}{{{{{\rm{c}}}}}}}+{I}_{{{{{{\rm{CO}}}}}}2}$$

Among *I*_indirect_, *I*_PD_, *I*_c–c_, and *I*_CO2_, the three currents *I*_PD_, *I*_c–c_, and *I*_CO2_ require an applied potential to occur, meaning they will instantaneously become zero when the potential is no longer applied. Therefore, for our analysis in this work we will group them together as “other” oxidation current, *I*_other_ (Eq. (3)). In contrast, indirect oxidation, which uses NiOOH as a chemical oxidant, still occurs without applied potential until NiOOH is completely converted to Ni(OH)_2_ (the removal of the potential will only stop electrochemical regeneration of NiOOH from Ni(OH)_2_). Our rate deconvolution method first measures a steady-state *I*_total_ at any given potential (i.e., 1.52 V_RHE_) and then stops applying the potential to measure the rate of indirect oxidation under open circuit conditions. This rate is measured by determining the consumption of the positive charge stored in NiOOH (*Q*) as a function of time (*t*) under the open circuit condition. Varying *t* and fitting a *Q* vs. *t* plot allows for extrapolation of the rate of indirect oxidation at *t* = 0. Since *t* = 0 under the open circuit condition is equivalent to the steady-state condition at 1.52 V_RHE_ (i.e., before removing the potential), *I*_indirect_ at 1.52 V_RHE_ can be obtained. Then, *I*_other_ is obtained by subtracting *I*_indirect_ from *I*_total_ (Eq. (4)).

3 $${I}_{{{{{{\rm{other}}}}}}}={I}_{{{{{{\rm{PD}}}}}}}+{I}_{{{{{{\rm{c}}}}}}-{{{{{\rm{c}}}}}}}+{I}_{{{{{{\rm{CO}}}}}}2}$$

4 $${I}_{{{{{{\rm{other}}}}}}}={I}_{{{{{{\rm{total}}}}}}}+{I}_{{{{{{\rm{indirect}}}}}}}$$

The results obtained from our rate deconvolution analysis are shown in Fig. 4c. As the pH decreases, *I*_total_ decreases but the pH-independent *I*_indirect_ remains approximately the same. As a result, the contribution from *I*_indirect_ to *I*_total_ (i.e., the percent *I*_indirect_) increases from 21% at pH 12 to 75% at pH 9. This results in more charge being used for indirect oxidation and thus for DHA production with decreasing pH since the data shown in Fig. 4a, b were obtained after passing the same total charge under each pH condition. The clear correlation between pH, the percent *I*_indirect_, and the amount of DHA produced indicates that PD oxidation is indeed suppressed as pH decreases and that indirect oxidation promotes secondary alcohol oxidation. The rate deconvolution results also agree well with the qualitative analysis of *I*_indirect_ and *I*_PD_ we made above using the LSV results, with the caveat that the current feature we assigned to *I*_PD_ also contains contributions from *I*_c–c_ and *I*_CO2_ for the case of glycerol oxidation.

Given the promising trend of increased DHA production with lower pH that was observed, we tested the effect of lowering the pH further to 8. The initial current density at pH 8, however, was significantly lower (Supplementary Fig. 11). Also, while a considerable amount of DHA was produced in the beginning of the electrolysis, the current density as well as the FE for DHA rapidly decreased (Supplementary Fig. 11) and the NiOOH film became deactivated. Thus, pH 8 did not appear to be a viable condition to perform glycerol oxidation using our NiOOH electrodes.

### Applied potential effects on product distribution and mechanism

**Fig. 5:**
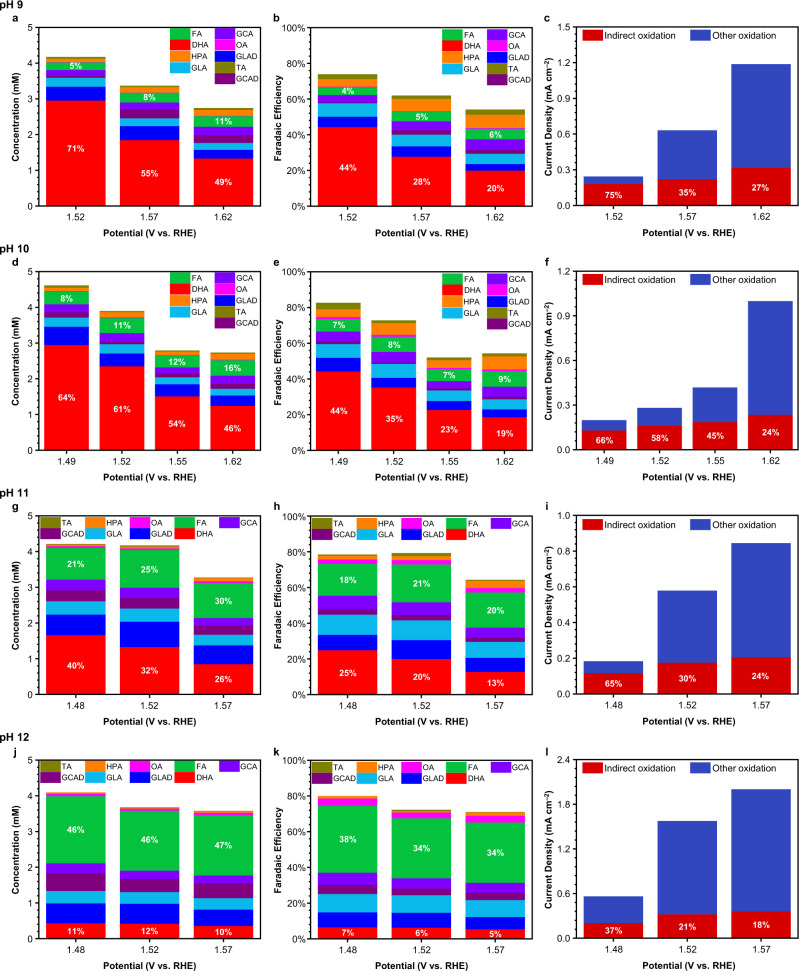
Glycerol oxidation results at pH 9-12 under various potential conditions. **a**, **d**, **g**, **j** Product distributions showing concentrations after passing 18 C at a constant potential. The white percentages indicate the relative selectivities for DHA (red) and FA (green). **b**, **e**, **h**, **k** Faradaic efficiencies (FEs) after passing 18 C. The percentages in white indicate the FEs for DHA (red) and FA (green). **c**, **f**, **i**, **l** Current density from *I*_indirect_ (red, percentage shown in white) and *I*_other_ (blue) oxidation of glycerol under various potential conditions. TA = tartronic acid, gold, HPA = hydroxypyruvic acid, orange, OA = oxalic acid, pink, FA = formic acid, green, GCA = glycolic acid, bright purple, GCAD = glycolaldehyde, dark purple, GLA = glyceric acid, light blue, GLAD = glyceraldehyde, dark blue, DHA = 1,3-dihydroxyacetone, red.

Separate from pH, the applied potential is also a key factor that affects the contributions from *I*_indirect_ and *I*_PD_^37^. In addition, it would be highly beneficial to elucidate the effect of potential on *I*_c–c_ and *I*_CO2_ at any given pH. Thus, we systematically varied the potential in pH 9-12 solutions and performed electrolysis and rate deconvolution experiments for each condition. The results obtained after passing 18 C are summarized in Fig. 5 with results obtained at other points during electrolysis at each condition compiled in Supplementary Figs. 4–8. The results in Fig. 5 offer a comprehensive and systematic understanding of the effects of the potential and pH on the complicated set of glycerol oxidation reactions. First examining the results obtained at pH 9, it is clear that the potential has a direct impact on DHA production, with more DHA produced at less positive potentials (Fig. 5a, b). This is as expected given our observation that the percent *I*_indirect_ increases as the potential decreases (Fig. 5c). The amount of FA produced does not vary much with the potential; however, the sum of the FEs for all detectable oxidation products decreases as the potential increases. As all potentials used in this experiment are not positive enough to induce meaningful OER currents and the increase in potential did not produce new unidentifiable solution products, the decrease in the sum of the FEs indicates that more CO_2_ is formed as the potential increases, meaning *I*_CO2_ increases with the potential (Supplementary Fig. 10b). As CO_2_ formation consumes FA, the fact that the amount of detected FA does not vary much as potential increases despite CO_2_ formation increasing indirectly suggests that C–C cleavage reactions, which are needed to form FA, are also enhanced with increased potential.

Similar potential effects were observed at pH 10-12 for the production of DHA, FA and CO_2_ (Fig. 5d–l). However, we note that the potential-dependence of DHA production becomes less pronounced as pH increases. This is partly because the potential-dependence of the percent *I*_indirect_ becomes less pronounced as pH increases (Fig. 5c,f,i,l) and partly because increasing the pH promotes C–C cleavage reactions, which is evidenced by the greater production of FA. This enhancement of C–C cleavage reactions means that in more basic conditions, C3 products such as DHA will be readily overoxidized to C2 and C1 products. The overoxidation of DHA, for example, will occur to a greater extent at higher DHA concentrations, meaning that even if more DHA is initially produced at less positive potentials, this greater accumulation of DHA will cause it to be consumed faster, decreasing the apparent potential dependence of DHA production under more basic conditions. Due to these reasons, the potential dependence of DHA production at pH 12 appears small (Fig. 5j, k).

In summary, we produced DHA as the major oxidation product on NiOOH electrodes with significant selectivity. As secondary alcohols are less reactive, it is more intuitive to think that their oxidation would require harsher conditions than those used for primary alcohol oxidation. However, by recognizing the presence of two dehydrogenation mechanisms on NiOOH and their mechanistic differences, we were able to predict that employing milder pH and potential conditions would enhance secondary alcohol oxidation. By systematically analyzing the impacts of pH and potential on C–C cleavage and CO_2_ formation reactions as well as on indirect and PD oxidation, this study provides a comprehensive and coherent understanding of the complicated set of glycerol oxidation reactions that occur on NiOOH, which will serve as a valuable mechanistic foundation to further study electrochemical glycerol valorization.

## Methods

### Materials

All chemicals used are commercially available and were used without further purification: glycerol (≥99.5%, Sigma-Aldrich), KOH (≥85%, Sigma-Aldrich), Ni(NO_3_)_2_•6H_2_O (Sigma-Aldrich), KNO_3_ (99%, Alfa Aesar), H_3_BO_3_ (99.8%, Alfa Aesar), K_2_SO_4_ (≥99.0%, Sigma-Aldrich), 1,3-dihydroxyacetone (Sigma-Aldrich, pharmaceutical standard), DL-glyceraldehyde (≥90%, Sigma-Aldrich), DL-glyceric acid (20% in water, TCI), glycolic acid (99%, Sigma-Aldrich), glycolaldehyde dimer (Sigma-Aldrich), oxalic acid (99.999%, Sigma-Aldrich), tartronic acid (97%, Thermo Scientific), sodium β-hydroxypyruvate hydrate (≥97.0%, Sigma-Aldrich), sodium mesoxalate monohydrate (≥98.0%, Sigma-Aldrich), sodium formate (≥99.0%, Sigma-Aldrich). Deionized water (Barnstead E-pure water purification system, resistivity >18 MΩ cm) was used to prepare all solutions.

### Ni(OH)_2_ electrode preparation

The amorphous α-Ni(OH)_2_ films were prepared using an established method to produce amorphous α-Ni(OH)_2_ films in which nitrate is electrochemically reduced, increasing the local pH at the WE and causing Ni(OH)_2_ to precipitate out as a film^44^. This reaction was controlled using a VMP2 multichannel potentiostat (Princeton Applied Research) and was conducted with a three-electrode setup in a single compartment glass cell using a fluorine-doped tin oxide (FTO) WE, Pt counter electrode (CE), and Ag/AgCl (4 M KCl) reference electrode (RE). The FTO WEs were prepared by washing large FTO plates (TEC15, 12–14 Ω sq^–1^ resistance, Hartford Glass) with soapy water, rinsing with DI water, rinsing with acetone, rinsing with isopropanol, and finally rinsing with >18 MΩ cm water. The washed FTO plates were then cut into 2.5 cm × 1 cm strips (thin films) or 2.5 cm × 1.25 cm strips (thick films), attaching Cu tape on top to provide electrical contact, and masking the FTO with electroplating tape (3 M Company) with one (thin films) or two (thick films) 0.5 cm^2^ holes punched out of it. The Pt CEs were prepared by sputter coating a 20 nm Ti adhesion layer followed by 100 nm of Pt on precleaned glass slides. The Ni(OH)_2_ films were then deposited from an aqueous plating solution of 10 mM Ni(NO_3_)_2_·6H_2_O and 30 mM KNO_3_ by galvanostatically maintaining a current of –0.25 mA cm^–2^ for 45 s (thin films) or a current of –0.37 mA cm^–2^ for 3 min (thick films). The resulting Ni(OH)_2_ films were rinsed with >18 MΩ cm water and dried with an air stream before further use. The thin films were used for LSV and rate deconvolution experiments while the thick films were used for constant potential electrolysis experiments. The thin films used for quantitative current analysis had an average of 24.6 nmol of Ni (of which all is electrochemically active) per 0.5 cm^2^ film^38^. As the goal of this study is not to achieve the highest conversion rate, but instead to understand the effect of the oxidation conditions on the reaction mechanisms, minimizing the heterogeneity of the catalyst was critical. Thus, even the “thick” films were still composed of uniformly thin Ni(OH)_2_ films whose thickness was increased just enough to produce meaningful oxidation currents that allowed for the electrolysis experiments to be completed in a reasonable time period (<8 h).

### Buffer preparation

For pH 8-12 conditions, a borate-based buffer was used. Given the complexity of borate buffer (e.g., dimer, trimer, etc. formation)^45^, K_2_SO_4_ was added as a supporting electrolyte to equalize the concentration of K^+^ in an effort to maintain a similar ionic strength across each pH. The pH 8-12 buffers were prepared as follows: a 0.75 M H_3_BO_3_ solution in >18 MΩ cm water was adjusted to the desired pH with solid KOH pellets, monitored by a pH probe (Accumet Basic, AB15), before K_2_SO_4_ was added to ensure a final concentration of K^+^ of ~1 M. The pH was readjusted with solid KOH or solid H_3_BO_3_ as needed. This high concentration of buffer and supporting electrolyte was chosen to ensure effective buffering and high solution conductivity across a wide pH range 8–12 (see Supplementary Fig. 2 for examples of more dilute buffer conditions or no supporting electrolyte). For pH 13 conditions, a 0.1 M KOH solution in in >18 MΩ cm water was used. For pH 13 conditions with added borate, a 0.75 M H_3_BO_3_ solution >18 MΩ cm water was adjusted to pH 13 with solid KOH. After adding glycerol or another organic species to the electrolyte solutions, which was observed to decrease the solution pH in most cases, the pH was adjusted again to the desired value by adding solid KOH.

### Electrochemical experiments

All electrochemical experiments were performed without iR-compensation. Linear sweep voltammetry (LSV) was performed with a three-electrode setup in an undivided glass cell. Ni(OH)_2_ thin films were used as the WE, Ag/AgCl (4 M KCl) was used as the RE for pH 8-12 conditions, Hg/HgO was used as the RE for pH 13 conditions, and the aforementioned sputter coated Pt electrode was used as the CE. The potential was swept from the open circuit in the positive direction at a scan rate of 10 mV s^–1^. Two CV cycles were completed for each film in blank buffer and with 10 mM of added organic species.

Electrolysis experiments were performed with a three-electrode setup in a divided glass H-cell with 14 mL of solution in each compartment. These compartments were loosely covered with parafilm. Four thick Ni(OH)_2_ films were used as the WE (connected with Cu tape), Ag/AgCl (4 M KCl) was used as the RE for pH 8-12 conditions, Hg/HgO (1 M NaOH) was used as the RE for pH 13 conditions, and Pt mesh was used as the CE. Electrolysis was performed by applying the desired potential (range of 1.48 V_RHE_–1.62 V_RHE_, depending on the pH (8-13)) potentiostatically to a rapidly stirred 25 mM solution of the organic species until specific amounts of charge had been passed. Powdered KOH(s) was periodically (every ~9 C) added to the WE compartment during electrolysis experiments carried out at pH 8-12 to help maintain a constant pH as the oxidation reaction consumes OH^–^ ions, lowering the pH.

For glycerol electrolysis, the total amount of charge passed was at least 36 C. This corresponds to the passage of ~53% of the charge required for a two-electron oxidation (i.e., glycerol oxidation to DHA) of all 25 mM glycerol in solution. In all cases the electrolysis time was <8 h. The amount of charge passed during the electrolysis experiments was limited as we want to get the best picture of product selectivities before additional over-oxidation of C3 and C2 products begins to occur more substantially and also to minimize any possible complications due to a long electrolysis time (>8 h). For electrolysis conditions that generated significantly higher oxidation currents, a total of up to 67.54 C were passed (i.e., the stoichiometric charge to convert all glycerol to DHA). For electrolysis of glycerol at pH 8, only 18 C were passed over ~8 h.

### Conversion from Ag/AgCl or Hg/HgO to the reversible hydrogen electrode (RHE)

The REs used for all the electrochemical experiments in this work were either Ag/AgCl (4 M KCl) or Hg/HgO. To allow for easier comparison across pHs, the potentials were converted to the RHE scale according to Eq. (5) where *E*_ref_ is the applied potential vs. the RE, *E*_RHE_ is the potential vs. RHE, *E*_ref_(NHE) is the potential of the RE vs. the normal hydrogen electrode (NHE), and pH is the pH of the solution. For Ag/AgCl, *E*_ref_(NHE) = 0.197 V. For Hg/HgO, *E*_ref_(NHE) = 0.141 V.

5 $${E}_{{{{{{\rm{RHE}}}}}}}={E}_{{{{{{\rm{ref}}}}}}}+{E}_{{{{{{\rm{ref}}}}}}}({{{{{\rm{NHE}}}}}})+0.0591\,{{{{{\rm{V}}}}}}\times {{{{{\rm{pH}}}}}}$$

### Product analysis

Quantification of electrolysis products was achieved using high-performance liquid chromatography (HPLC, Prominence-i LC 2030 C 3D, Shimadzu). The mobile phase was 0.1% H_2_SO_4_ in >18 MΩ cm water and the stationary phase was an ICSep ICE-COREGEL 87H3 column. The flow rate was 0.5 mL min^–1^ with a column temperature of 40 °C. Integration of PDA absorbances at multiple wavelengths were compared to calibration curves of all identified products to quantify each product. Quantification of co-eluting peaks was done using unique absorbances and/or solving a system of equations using the integration from multiple wavelengths. Aliquots (150 μL) were taken after approximately 3.25 C, 9 C, 18 C, 27 C, 36 C, 45 C, 54 C, and 67.54 C, depending on the length of the electrolysis. For the pH 13 glycerol electrolysis, aliquots were only collected after 9 C, 18 C, 36 C, 54 C and 67.54 C. For electrolysis of FA, a single aliquot was analyzed after 9 C. For electrolysis of DHA at pH 8, aliquots were analyzed after 3.25 C and 5 C.

Relative selectivities for each product were calculated using Eq. (1) as described above. Ideally, selectivity in glycerol oxidation, which can involve C–C cleavage reactions, would be determined using Eq. (6), where the numerator is divided by *n*, the stoichiometric coefficient in the balanced reaction to form a specific product from 1 glycerol molecule (e.g., for C3, C2, or C1 products, *n* = 1, 3/2, or 3, respectively)^6^. However, we were unable to accurately quantify glycerol consumption due to poor separation of glycerol and DHA by HPLC and lack of a unique absorbance for glycerol (see discussion in the next paragraph). Thus, we used Eq. (1) above to discuss the relative selectivities. We note, however, that as n is not considered in Eq. (1), the selectivity for C3 products is underestimated compared to the absolute selectivities that would be obtained from Eq. (6). Nonetheless, we believe that reporting these relative selectivities alongside absolute product concentrations and FEs is the best practice for reporting complicated glycerol oxidation results involving C–C cleavage reactions and variable C count products when the consumption of glycerol cannot be accurately quantified.

6 $${{{{{\rm{Absolute}}}}}}\,{{{{{\rm{Selectivity}}}}}}\,\left(\%\right)=\frac{({{{{{\rm{mol}}}}}}\; {{{{{\rm{of}}}}}}\; {{{{{\rm{specific}}}}}}\; {{{{{\rm{product}}}}}})/{{{{{\rm{n}}}}}}}{{{{{{\rm{mol}}}}}}\; {{{{{\rm{of}}}}}}\; {{{{{\rm{consumed}}}}}}\; {{{{{\rm{reactant}}}}}}}\, \times 100\%$$

With our HPLC setup and method, the elution peaks for glycerol and DHA overlap significantly. This separation could not be improved with variations in the HPLC method parameters. DHA has a unique absorbance at 270 nm, as well as significant absorbance at 205 nm and 215 nm, and so could be accurately quantified by integrating the peak area detected by the PDA detector at those wavelengths. However, glycerol has only a trailing UV absorbance which was only intense enough to be quantified using the peak area at 190 nm. Unfortunately, DHA also absorbs at 190 nm, much more intensely than glycerol, which convolutes attempts to quantify glycerol at 190 nm due to overlap with DHA. Even our best efforts to use the separately determined concentration of DHA to correct for peak convolutions at 190 nm were unsuccessful.

Faradaic efficiencies (FEs) for each product were calculated using Supplementary Eq. (4) as described in the Supplementary Methods.

### Rate deconvolution procedure

While a detailed explanation of our three-step rate deconvolution procedure is provided in our previous work^38,41^, a sufficient description and overview of the process will be provided here. Rate deconvolution was done for all tested potentials at pH 9-12. The procedure was performed with 30 mL of rapidly stirred 25 mM glycerol solutions in a single cell with a three-electrode setup. The WE was a thin Ni(OH)_2_ film, the RE was Ag/AgCl (4 M KCl), and the CE was platinum mesh. Prior to use for the rate deconvolution experiments, each Ni(OH)_2_ film was tested using CV (two cycles starting at OCP) to confirm that the Ni(OH)_2_/NiOOH peak and water oxidation behavior of each film were consistent.

In the first step of the three-step procedure, the desired potential (range of 1.48 V_RHE_–1.62 V_RHE_, depending on the pH (9-12)) was applied potentiostatically to the WE in a rapidly stirred solution long enough for the current to stabilize to an approximately steady-state value (105 s at pH 9, 90 s at pH 10 and pH 11, 75 s at pH 12). This converts the film into the steady-state condition it would be in during a potentiostatic electrolysis at a given applied potential, and during this step, all oxidation pathways are active, including the indirect oxidation, PD oxidation, C–C cleavage, and CO_2_ generation pathways. During the second step, the potential is no longer applied and the film is allowed to sit in the stirred solution under open circuit conditions. During this time, the PD, C–C cleavage, and CO_2_ generation pathways cannot occur, and neither can reoxidation of Ni(OH)_2_, as all of these processes require applied potential; however, the indirect pathway, which proceeds through a chemical (rather than electrochemical) HAT step, still occurs, reducing higher valent Ni sites back to Ni(OH)_2_. During the third step, the remaining higher valent sites still left after step 2 are rapidly reduced back to Ni(OH)_2_ by sweeping the potential from open circuit to 0.1 V (pH 9) or 0 V (pH 10-12) vs. Ag/AgCl at a scan rate of 1 V s^–1^ and then holding the potential at 0.1 V (pH 9) or 0 V (pH 10-12) for 20 s until all remaining high valent Ni is reduced to Ni(OH)_2_. The magnitude of the charge passed during this third, reductive step corresponds to the amount of charge that was still stored in the film after some of it was used to oxidize the organic substrate through the indirect pathway in step 2 during the time (*t*) at open circuit.

By repeating this whole three-step process with different times stirring at open circuit in step 2, we can construct plots showing the disappearance of charge (*Q*) from the film as a function of *t*. This data can be analyzed to determine the instantaneous rate of indirect oxidation at *t* = 0, which corresponds to the rate of the indirect pathway under electrolysis conditions at the applied potential in step 1. As this rate is a measure of *Q* vs. *t* and will be in units of C s^–1^ (equivalent to units of A), this rate directly corresponds to the current (*I*_indirect_) used for indirect oxidation under electrolysis conditions at the applied potential in step 1.

The *Q* vs. *t* data are first analyzed to determine the kinetic dependence of the reaction rate on *Q*. Three common reaction orders and their integrated rate laws from general chemistry were considered: zeroth, first, and second order. For a zeroth order dependence, the *Q* vs. *t* plot should be linear. For a first order dependence, a ln(*Q*) vs. *t* plot should be linear. For a second order dependence, a *Q*^–1^ vs. *t* plot should be linear. In this study, the *Q*^–1^ vs. *t* plots were linear (Supplementary Figs. 12–15), indicating an apparent second order dependence on the charge stored in the NiOOH film, consistent with previous studies of aliphatic alcohol oxidation^38^. We note that this apparent second order dependence on charge stored in the NiOOH is likely over-simplified and the “true” rate equation for indirect oxidation may be more complicated. However, for this rate deconvolution we do not need the “true” rate equation for the reaction and instead only require that our model accurately describes the change in charge stored in the NiOOH film over the timespan of interest. This apparent second order dependence does so nicely. The linear fit of the *Q*^–1^ vs. *t* plots will give the pseudo-second order rate constant, *k*_obs_, for the indirect oxidation reaction as the slope of the linear fit and the inverse of the amount of charge, *Q*_0_^–1^, stored in the film at *t* = 0 under open circuit conditions as the intercept of the linear fit (Eq. (7)). We obtain the pseudo-second order *k*_obs_ that reflects the true rate constant for indirect oxidation, *k*_ind_, because our rapid stirring of the solutions in this procedure ensures constant concentrations of the other reactants (i.e., glycerol and OH^–^ ions, with reaction orders a and b, respectively) at the WE surface so that we don’t have to consider their effect on the reaction rate (Eq. (8)). Finally, we can use *k*_obs_ and *Q*_0_ from the linear fits to determine the instantaneous rate at *t* = 0 (*I*_indirect_) according to the second order rate equation shown in Eq. (9).

7 $$\frac{1}{{Q}_{t}}={k}_{{{{{{\rm{obs}}}}}}}(t)+\frac{1}{{Q}_{0}}$$

8 $${k}_{{{{{{\rm{obs}}}}}}}={k}_{{{{{{\rm{ind}}}}}}}\bullet {[{{{{{\rm{glycerol}}}}}}]}^{a}\bullet {[{{{{{{\rm{OH}}}}}}}^{-}]}^{b}$$

9 $${I}_{{{{{{\rm{indirect}}}}}}}={{{{{\rm{Rate}}}}}}\,\left(t=0\right)={k}_{{{{{{\rm{obs}}}}}}}\bullet ({{Q}_{0}})^{2}$$

When conducting the rate deconvolution trials for a given condition, the three-step process was repeated four separate times using four different Ni(OH)_2_ films for each length of time tested at open circuit in step 2, with the results being averaged to get the data points shown in the *Q*^–1^ vs. *t* plots in Supplementary Figs. 12–15. When conducting these trials, the Ni(OH)_2_ films were replaced after being used to collect no more than four data points. In addition, the glycerol solution was replaced with a fresh 30 mL solution each time the Ni(OH)_2_ WE was replaced. We have also developed a procedure to monitor and correct for small variations in the active Ni content from film to film and any slight decrease in the total active Ni sites in each film that may occur from repeated use, which is described in detail in the Supplementary Methods. This procedure ensured that these variations were properly accounted for so as not to affect our measurements and ensure that accurate and reliable rate deconvolution results could be obtained.

Rate deconvolution could not be carried out at pH 8 due to insufficient charge stored in the film given that at this pH, 1.52 V_RHE_ is too far before the Ni(OH)_2_/NiOOH oxidation peak to charge the film significantly. Similarly, rate deconvolution could not be carried out at pH 13 due to insufficient charge stored in the film. In this case, the average valence of Ni was kept low due to rapid consumption of charge by PD oxidation and C–C cleavage reactions.

## Supplementary information

Supplementary Information

## Data Availability

All data are available in the paper or the supplementary materials. In addition, the source data used to construct the plots shown in the paper and Supplementary Materials are provided with this paper. Source data are provided with this paper.

## Source data

Source Data
