# Supplementary material for: Predictive control of selective secondary alcohol oxidation of glycerol on NiOOH
Source: Nat Commun. 2022 Oct 4;13:5848. doi: 10.1038/s41467-022-33637-7 (PMC9532427; doi:10.1038/s41467-022-33637-7)
Supplement: Supplementary file 1 — Supplementary Information [file 41467_2022_33637_MOESM1_ESM.pdf]

Supplementary Materials

Predictive Control of Selective Secondary Alcohol Oxidation of Glycerol on NiOOH

McKenna K. Goetz, Michael T. Bender, and Kyoung-Shin Choi

Department of Chemistry, University of Wisconsin-Madison, Madison, WI 53706, USA

*Correspondence to: kschoi@chem.wisc.edu

Table of Contents

Supplementary Methods ... **3**
Mathematical adjustment of measured charge values ... 3
Reasons for the mathematical calibration of the measured charge values. ... 3
Procedure to account for film deactivation during data collection. ... 3
Procedure to account for the differences in the amount of active Ni from film to film. ... 3
The mathematical calibration equations. ... 4
Faradaic Efficiency Calculations ... 4
Determination of e^- counts for various products formed during glycerol electrooxidation ... 4
Alternative method for determining e^- counts for C2 products. ... 5
Supplementary Table 1. Summary of electron counts used in the calculations of the FEs ... 6
Supplementary References ... **6**
Supplementary Figures and Tables ... **7**
Supplementary Fig. 1. Decomposition of DHA, GLAD, and GCAD in 0.1 M KOH (pH 13) ... 7
Supplementary Table 2. Stability of DHA and GLAD at pH 12 ... 7
Supplementary Fig. 2. CVs for buffer optimization ... 8
Supplementary Fig. 3. Electrolysis of 25 mM glycerol at low applied potential ... 9
Supplementary Fig. 4. Glycerol oxidation results at pH 9 during electrolysis ... 10
Supplementary Fig. 5. Glycerol oxidation results at pH 10 during electrolysis ... 11
Supplementary Fig. 6. Glycerol oxidation results at pH 11 during electrolysis ... 12
Supplementary Fig. 7. Glycerol oxidation results at pH 12 during electrolysis ... 13
Supplementary Fig. 8. Glycerol oxidation results at pH 13 during electrolysis ... 14
Supplementary Fig. 9. Glycerol oxidation results at pH 13 with added borate ... 14
Supplementary Fig. 10. FA oxidation results under various potential and pH conditions ... 15
Supplementary Fig. 11. Glycerol oxidation results at pH 8 during electrolysis ... 15
Supplementary Fig. 12. Q^{-1} vs t plots for rate deconvolution experiments at pH 9 ... 16
Supplementary Fig. 13. Q^{-1} vs t plots for rate deconvolution experiments at pH 10 ... 17
Supplementary Fig. 14. Q^{-1} vs t plots for rate deconvolution experiments at pH 11 ... 18
Supplementary Fig. 15. Q^{-1} vs t plots for rate deconvolution experiments at pH 12 ... 19

Supplementary Methods

Mathematical adjustment of measured charge values[1]

Reasons for the mathematical calibration of the measured charge values.

The number of active Ni sites in the as deposited α-$Ni(OH)_2$ films can vary slightly from film to film. Additionally, the ability to store charge in the films often decreases gradually when a film is repeatedly used to perform the three-step rate deconvolution procedure. We will refer to this decrease as deactivation and note that it is likely due to the rapid reduction in the third step of the procedure. Because of these factors, the accuracy of our analysis can be improved by mathematically adjusting the measured charge stored in the $Ni(OH)_2$ films to account for these differences and changes. This ensures that for a given reaction condition, the differences in the reported charge stored in the films are due only to the differences in the time they spent at open circuit (i.e. reaction time) and not to differences in the initial amount of active Ni from film to film or to the films having undergone differing degrees of deactivation from repeated use.

Procedure to account for film deactivation during data collection.

When conducting our rate deconvolution trials, each film is used to collect four data points. This means they were used to perform the three-step procedure four separate times with the time in step 2 varied for each instance. After collecting each of these four data points, we immediately performed an additional instance of the three-step procedure (referred to as a calibration three-step procedure) in the same solution with 0.1 s chosen as the time at open circuit conditions in step 2. The charge passed when reducing the film back to $Ni(OH)_2$ in step 3 for this calibration three-step procedure is referred to as $Q_{cal(t = 0.1)}$. Accordingly, each $Ni(OH)_2$ film was used to perform 8 three-step procedure measurements, four for data collection and four for calibration. If the film did not undergo deactivation during the data measurements, the four $Q_{cal(t = 0.1)}$ measurements would be identical. If they decrease, however, a degree of film deactivation is implied. In that case, the $Q_{cal(t = 0.1)}$ measurements can be used to mathematically adjust the data measurements to compensate for differences in the degree of film deactivation using the procedure outlined below.

We note that 0.1 s was used as the time at open circuit in step 2 for the calibration three-step procedures rather than the more intuitive 0 s because there was a measurement artifact present when evaluating the charge stored in the films at 0 s that made these measurements unreliable. The use of 0.1 s instead of 0 s should not influence the results of the calibration, however, because as long as the same 0.1 s was used for all the calibration measurements, this would still be an accurate way to compare differences in the amount of active Ni sites in the films.

Procedure to account for the differences in the initial amount of active Ni from film to film.

In addition to its use in correcting for any differences in film deactivation, the $Q_{cal(t = 0.1)}$ values can also be used to correct for any difference in the initial number of active Ni sites in our as deposited films. The $Q_{cal(t = 0.1)}$ value obtained in the first calibration three-step procedure taken for each $Ni(OH)_2$ film is proportional to the initial amount of active Ni sites in that film. As such, when collecting a rate deconvolution data set for each given reaction condition, we averaged the first $Q_{cal(t = 0.1)}$ values collected for each film to obtain $Q_{ave(t = 0.1)}$. We could then compare this $Q_{ave(t = 0.1)}$ to the $Q_{cal(t = 0.1)}$ value measured for each data point and, through the equations outlined below, use the difference to adjust the measured charge stored for a given data point to compensate for any variations in the number of active Ni sites from film to film or degree of deactivation from measurement to measurement. Doing this for all our data points yield adjusted values for the charge

stored that represents the value that would have been measured had all the films had the average initial number of active Ni sites and not yet undergone any deactivation, thereby removing any distorting effects on our data from differences in initial active Ni content or degree of film deactivation.

The mathematical calibration equations.

The equations used to perform the calibration described above depend on the reaction order with respect to the charge stored in the NiOOH film. For the glycerol oxidation reaction described here, the reaction is best modeled as second order with respect to the charge stored in the NiOOH film. Thus, the mathematical adjustment to the measured charge stored was done using Supplementary Equation (1) where $Q_{\mathrm{obs}(t)}$ is the magnitude of the charge passed in the third step of the rate deconvolution trial performed with the film stirring for t seconds at open circuit in step 2 and $Q_{\mathrm{adj}(t)}$ is the adjusted (i.e. calibrated) value of the charge passed in the third reductive step for the trial at t seconds at open circuit that accounts for any deviation between $Q_{\mathrm{ave}(t\,=\,0.1)}$ and $Q_{\mathrm{cal}(t\,=\,0.1)}$. The resulting values of $Q_{\mathrm{adj}(t)}$ are what we used to construct the Q^{-1} vs t plots shown in Supplementary Fig. 12-15.

$$\frac{1}{Q_{\mathrm{adj}(t)}} = \frac{1}{Q_{\mathrm{ave}(t\,=\,0.1)}} - \frac{1}{Q_{\mathrm{obs}(t\,=\,0.1)}} + \frac{1}{Q_{\mathrm{obs}(t)}} \tag{1}$$

Supplementary Equation (1) can be derived by noting that, over the timeframe of interest, the disappearance of the charge stored in the NiOOH film can be described according to Supplementary Equation (2), which is the pseudo-second order integrated rate law expressed in terms of coulombs.

$$\frac{1}{Q_{\mathrm{obs}(t)}} = \frac{1}{Q_0} + k_{\mathrm{obs}}t \tag{2}$$

From this, we can note that the difference between the observed charge passed in the reductive step and the value that would have been observed had the film started with the average number of active Ni sites after being oxidized in step 1 is given by Supplementary Equation (3), which can be rearranged to give Supplementary Equation (1) above.

$$\frac{1}{Q_{\mathrm{adj}(t)}} - \frac{1}{Q_{\mathrm{obs}(t)}} = \frac{1}{Q_{\mathrm{ave}(t\,=\,0.1)}} - \frac{1}{Q_{\mathrm{obs}(t\,=\,0.1)}} \tag{3}$$

Faradaic Efficiency Calculations

The Faradaic efficiencies (FEs) for the production of each product formed during glycerol electrooxidation were calculated using Supplementary Equation (4), where n is the moles formed of that product (determined by multiplying the concentration found by HPLC times the volume of the solution (14 mL)), z is the number of electrons required to form that product (Supplementary Table 1), F is Faraday's constant (96485 C mol^{-1}), and Q is the total charge passed.

$$\mathrm{FE}\ (\%) = \frac{n \cdot z \cdot F}{Q} \times 100\% \tag{4}$$

Determination of e^{-} counts for various products formed during glycerol electrooxidation.

For each possible product of glycerol electrooxidation that was observed in this work, the electrochemical oxidation half-reaction was balanced to determine the number of electrons required for that particular half-reaction. If the stoichiometric coefficient for a given product was

>1 in the balanced reaction (e.g., 1 glycerol will produce 3 FA), the number of electrons for that half-reaction was divided by that stoichiometric coefficient to give the number of electrons required to form 1 mole of that product. This number of electrons was then used in the FE calculation (Supplementary Equation (4)). The balanced reactions and electron count for each product are shown below and are summarized in Supplementary Table 1.

Glycerol → DHA

$C_3O_3H_8 + 2\ OH^- \rightarrow C_3O_3H_6 + 2\ H_2O + 2\ e^-$ $\qquad$ e^- count for DHA = $2\ e^-$

Glycerol → GLAD

$C_3O_3H_8 + 2\ OH^- \rightarrow C_3O_3H_6 + 2\ H_2O + 2\ e^-$ $\qquad$ e^- count for GLAD = $2\ e^-$

Glycerol → GLA

$C_3O_3H_8 + 4\ OH^- \rightarrow C_3O_4H_6 + 3\ H_2O + 4\ e^-$ $\qquad$ e^- count for GLA = $4\ e^-$

Glycerol → HPA

$C_3O_3H_8 + 6\ OH^- \rightarrow C_3O_4H_4 + 5\ H_2O + 6\ e^-$ $\qquad$ e^- count for HPA = $6\ e^-$

Glycerol → TA

$C_3O_3H_8 + 8\ OH^- \rightarrow C_3O_5H_4 + 6\ H_2O + 8\ e^-$ $\qquad$ e^- count for TA = $8\ e^-$

Glycerol → GCAD

$2\ C_3O_3H_8 + 4\ OH^- \rightarrow 3\ C_2O_2H_4 + 4\ H_2O + 4\ e^-$ $\qquad$ e^- count for GCAD = $4/3\ e^-$

Glycerol → GCA

$2\ C_3O_3H_8 + 10\ OH^- \rightarrow 3\ C_2O_3H_4 + 7\ H_2O + 10\ e^-$ $\qquad$ e^- count for GCA = $10/3\ e^-$

Glycerol → OA

$2\ C_3O_3H_8 + 22\ OH^- \rightarrow 3\ C_2O_4H_2 + 16\ H_2O + 22\ e^-$ $\qquad$ e^- count for OA = $22/3\ e^-$

Glycerol → FA

$C_3O_3H_8 + 8\ OH^- \rightarrow 3\ CO_2H_2 + 5\ H_2O + 8\ e^-$ $\qquad$ e^- count for FA = $8/3\ e^-$

Alternative method for determining e^- counts for C2 products.

For the C2 products, it is also possible to arrive at these electron counts by balancing half-reactions where glycerol is transformed in one C2 product and one FA molecule, and then subtracting the electrons required for FA ($8/3\ e^-$) to determine how many electrons can be attributed to the C2 product. These alternative half-reactions are shown below for each C2 product.

Glycerol → GCAD + FA

$C_3O_3H_8 + 4\ OH^- \rightarrow C_2O_2H_4 + CO_2H_2 + 3\ H_2O + 4\ e^-$

e^- count for GCAD = $4\ e^- - (8/3\ e^-) = 4/3\ e^-$

Glycerol → GCA + FA

$C_3O_3H_8 + 6\ OH^- \rightarrow C_2O_3H_4 + CO_2H_2 + 4\ H_2O + 6\ e^-$

e^- count for GCA = $6\ e^- - (8/3\ e^-) = 10/3\ e^-$

Glycerol → OA + FA

$C_3O_3H_8 + 10\ OH^- \rightarrow C_2O_4H_2 + CO_2H_2 + 7\ H_2O + 10\ e^-$

e^- count for OA = $10\ e^- - (8/3\ e^-) = 22/3\ e^-$

Supplementary Table 1. Summary of electron counts used in the calculations of the FEs for the production of each product of glycerol electrooxidation.

Compound	e^- count for FE calculations
DHA	2
GLAD	2
GLA	4
HPA	6
TA	8
GCAD	4/3
GCA	10/3
OA	22/3
FA	8/3

Supplementary References

1. Bender, M. T. & Choi, K.-S. Electrochemical Dehydrogenation Pathways of Amines to Nitriles on NiOOH. *JACS Au* **2**, 1169–1180 (2022).

Supplementary Figures and Tables

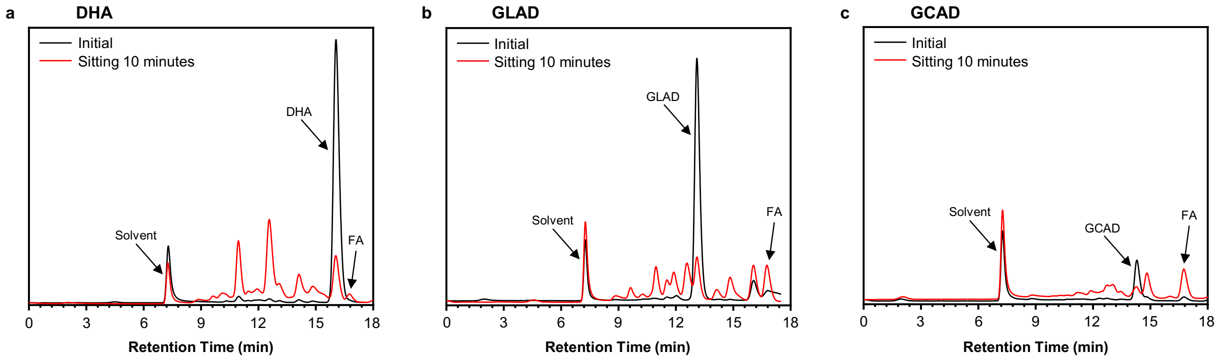

Supplementary Fig. 1. HPLC chromatographs at 205 nm showing the rapid decomposition of **a** DHA, **b** GLAD, and **c** GCAD in 0.1 M KOH (pH 13) solution. Solutions containing 25 mM of a given substrate were allowed to stir rapidly for 10 minutes before being neutralized with H_2SO_4 to stop decomposition and then analyzed by HPLC. Within just 10 minutes, >75% of the initial species (DHA, GLAD, or GCAD) had decomposed to formic acid and a multitude of products.

Supplementary Table 2. Concentrations of DHA and GLAD over time in separate pH 12 solutions (borate buffer) monitored by HPLC. A ~2 mM solution of DHA or ~5 mM solution of GLAD was prepared in pH 12 borate buffer and allowed to stir open to air. Aliquots were collected periodically and analyzed by HPLC. It was assumed the stability of DHA and GLAD in pH 12 solution would extend to lower pH conditions (8-11). The slight decrease in GLAD concentration was due to isomerization to DHA.

Time (min)	DHA (mM)	GLAD (mM)
0	1.61	5.51
35	1.61	5.45
70	1.60	5.48
105	1.61	5.37
140	1.62	5.38

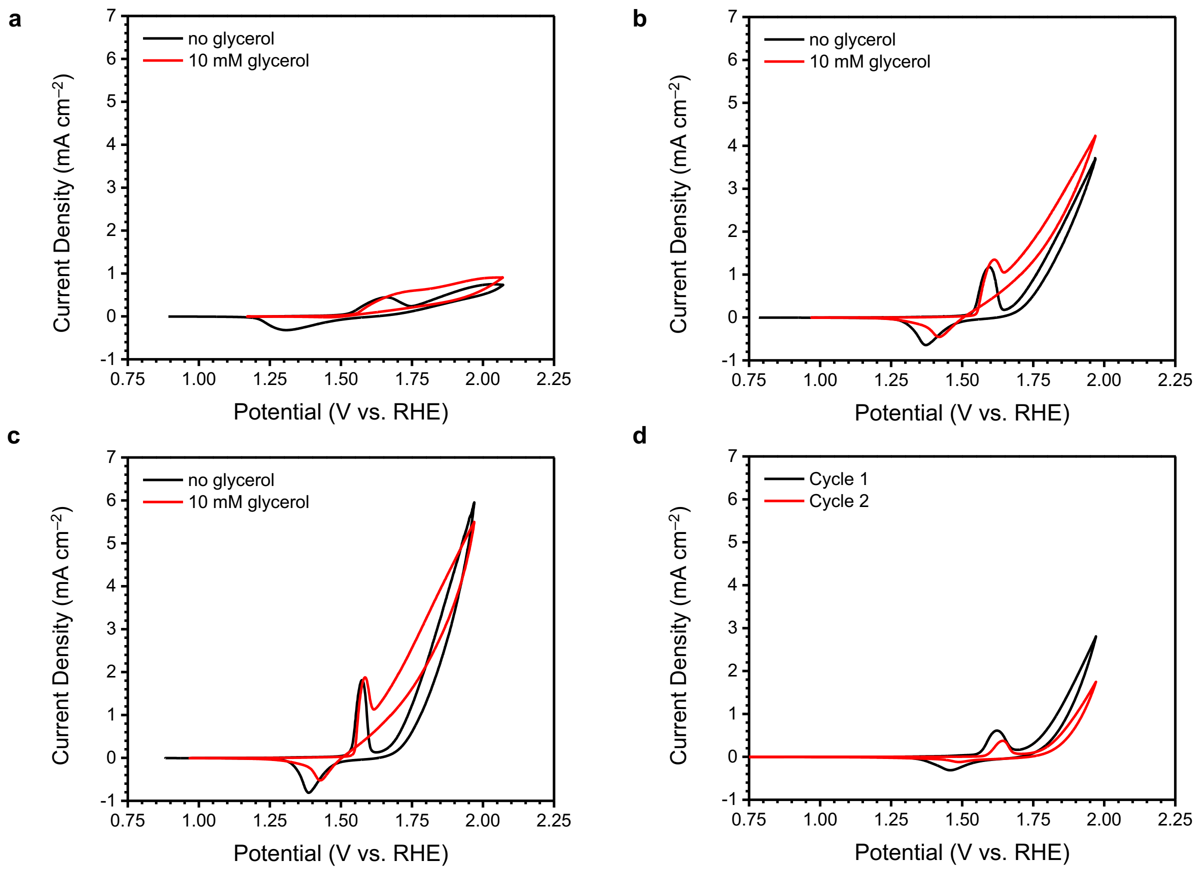

Supplementary Fig. 2. Cyclic voltammograms of $Ni(OH)_2$ films at pH 8 with **a** 0.1 M borate buffer without supporting electrolyte, **b** 0.75 M borate buffer without supporting electrolyte, **c** 0.75 M borate buffer with 0.41 M K_2SO_4 as a supporting electrolyte, and **d** 0.1 M sodium phosphate buffer. The Ni film itself is not stable in phosphate buffer **d**. In the presence of 10 mM glycerol, the Ni film is not stable if the buffering capacity is too low (e.g., in **a**) due to a rapid pH drop at the electrode surface from glycerol oxidation that overwhelms the buffer. The lower local pH induces the dissolution of Ni ions. Use of a supporting electrolyte increases solution conductivity giving a sharper CV that better represents the glycerol oxidation activity at a given potential (compare **b** and **c**).

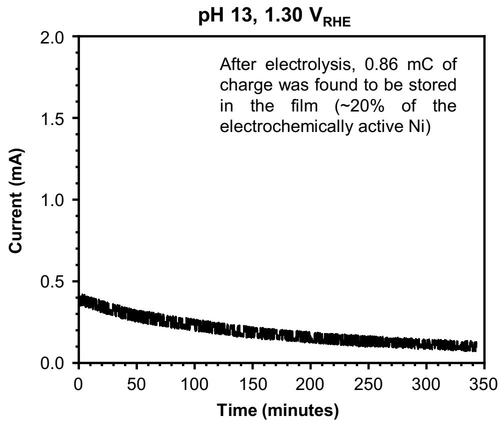

Supplementary Fig. 3. Current versus time profile for constant-potential oxidation of glycerol at 1.30 V_{RHE} in a pH 13 solution. This experiment examines whether the glycerol oxidation current shown in Fig. 3b (pH 13) that initiates around 1.30 V_{RHE} before the onset of the $Ni(OH)_2$/NiOOH peak is due to direct glycerol oxidation on $Ni(OH)_2$. Our result shows a non-negligible glycerol oxidation current with a correspondingly small conversion of glycerol; however, reduction of the films after electrolysis reveals that at 1.30 V_{RHE} a non-negligible amount of NiOOH is present, meaning glycerol oxidation at this potential is still due to NiOOH. We note that a small fraction of $Ni(OH)_2$ can be oxidized to NiOOH before the $Ni(OH)_2$/NiOOH peak due to the heterogeneity of the $Ni(OH)_2$ surface (i.e., Ni^{2+} in imperfect coordination environments can be oxidized more readily). While the oxidation current due to this fraction of Ni^{2+} is negligible without glycerol, when glycerol is present in strongly basic conditions (pH 13), the resulting high valent Ni ions can go through multiple catalytic cycles for glycerol oxidation during the LSV, generating a non-negligible oxidation current before the $Ni(OH)_2$/NiOOH peak as shown in Fig. 3b. We did not find any evidence that glycerol oxidation can occur directly on $Ni(OH)_2$. Glycerol oxidation before the $Ni(OH)_2$/NiOOH peak is not observed under lower pH conditions in Fig. 3 because the oxidation of $Ni(OH)_2$ and/or regeneration of NiOOH is more difficult and slower under lower pH conditions.

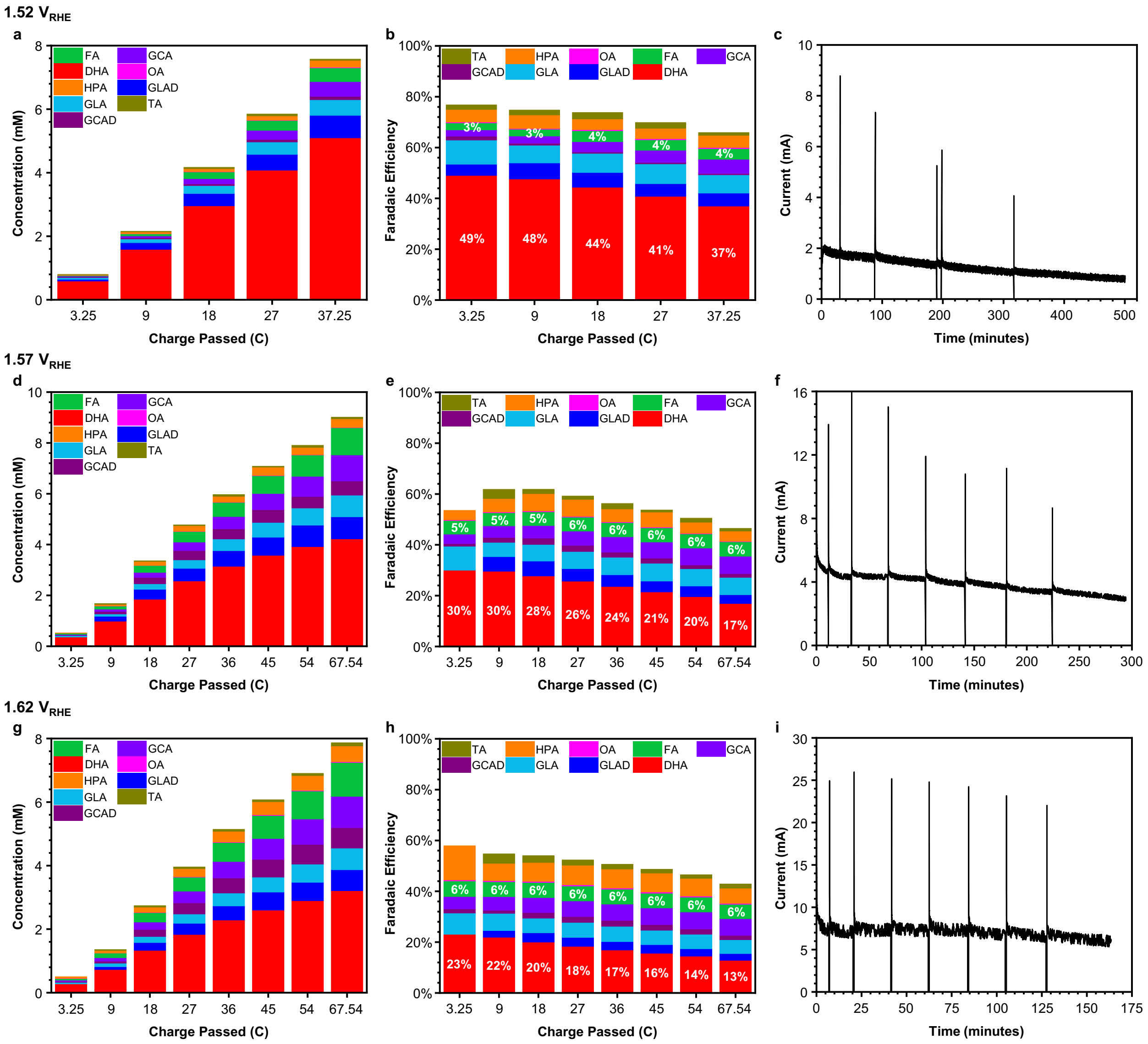

Supplementary Fig. 4. Glycerol oxidation results at pH 9 during electrolysis at a constant potential of 1.52 V_{RHE}, 1.57 V_{RHE}, or 1.62 V_{RHE}. **a, d, g** Product distributions showing product concentrations throughout electrolysis. **b, e, h** Faradaic efficiencies throughout electrolysis. The percentages in white indicate the specific FEs for DHA (red) and FA (green). **c, f, i** Current versus time profile for the electrolysis.

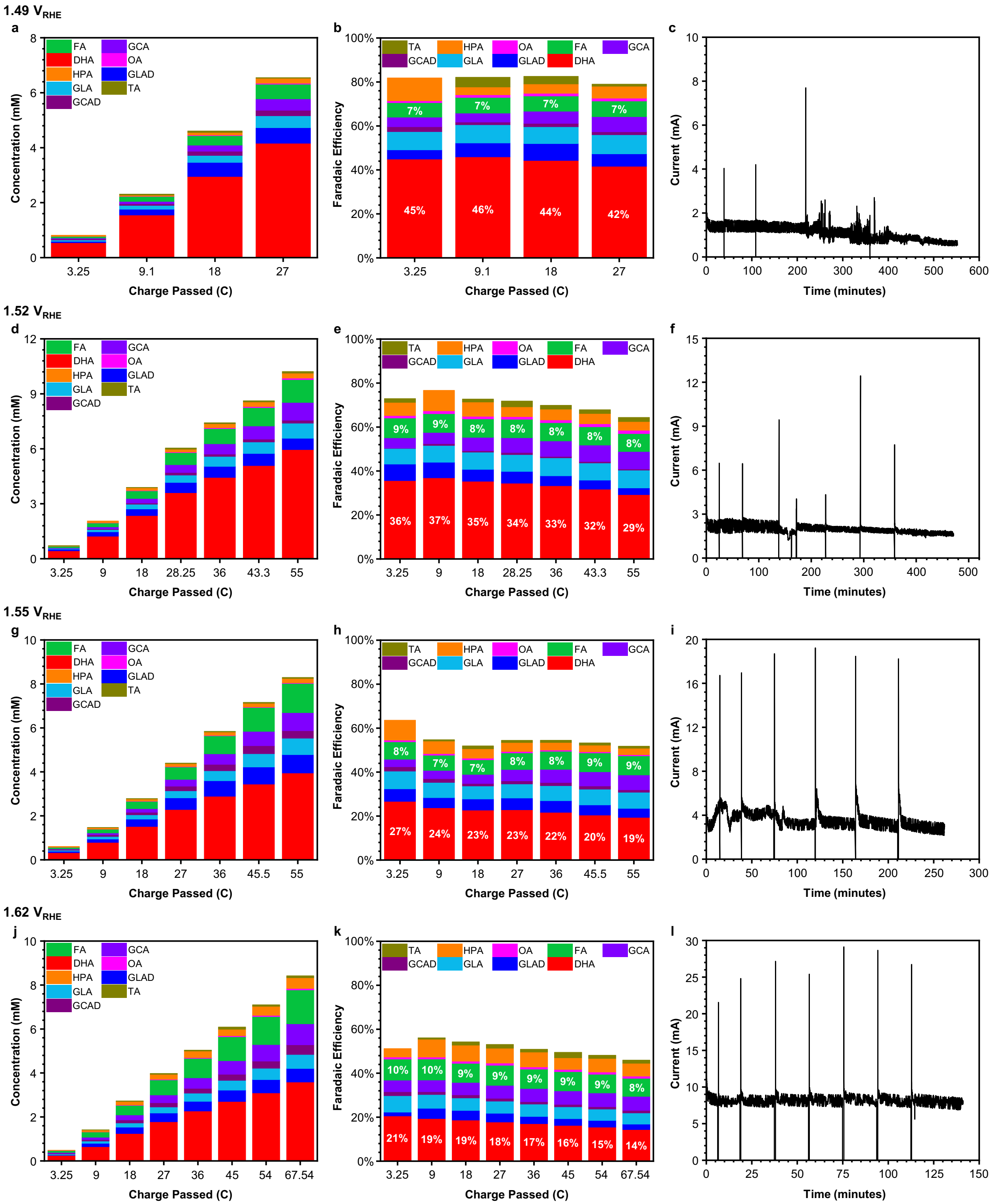

Supplementary Fig. 5. Glycerol oxidation results at pH 10 during electrolysis at a constant potential of 1.49 V_{RHE}, 1.52 V_{RHE}, 1.55 V_{RHE}, or 1.62 V_{RHE}. **a, d, g, j** Product distributions showing product concentrations throughout electrolysis. **b, e, h, k** Faradaic efficiencies throughout electrolysis. The percentages in white indicate the specific FEs for DHA (red) and FA (green). **c, f, i, l** Current versus time profile for the electrolysis.

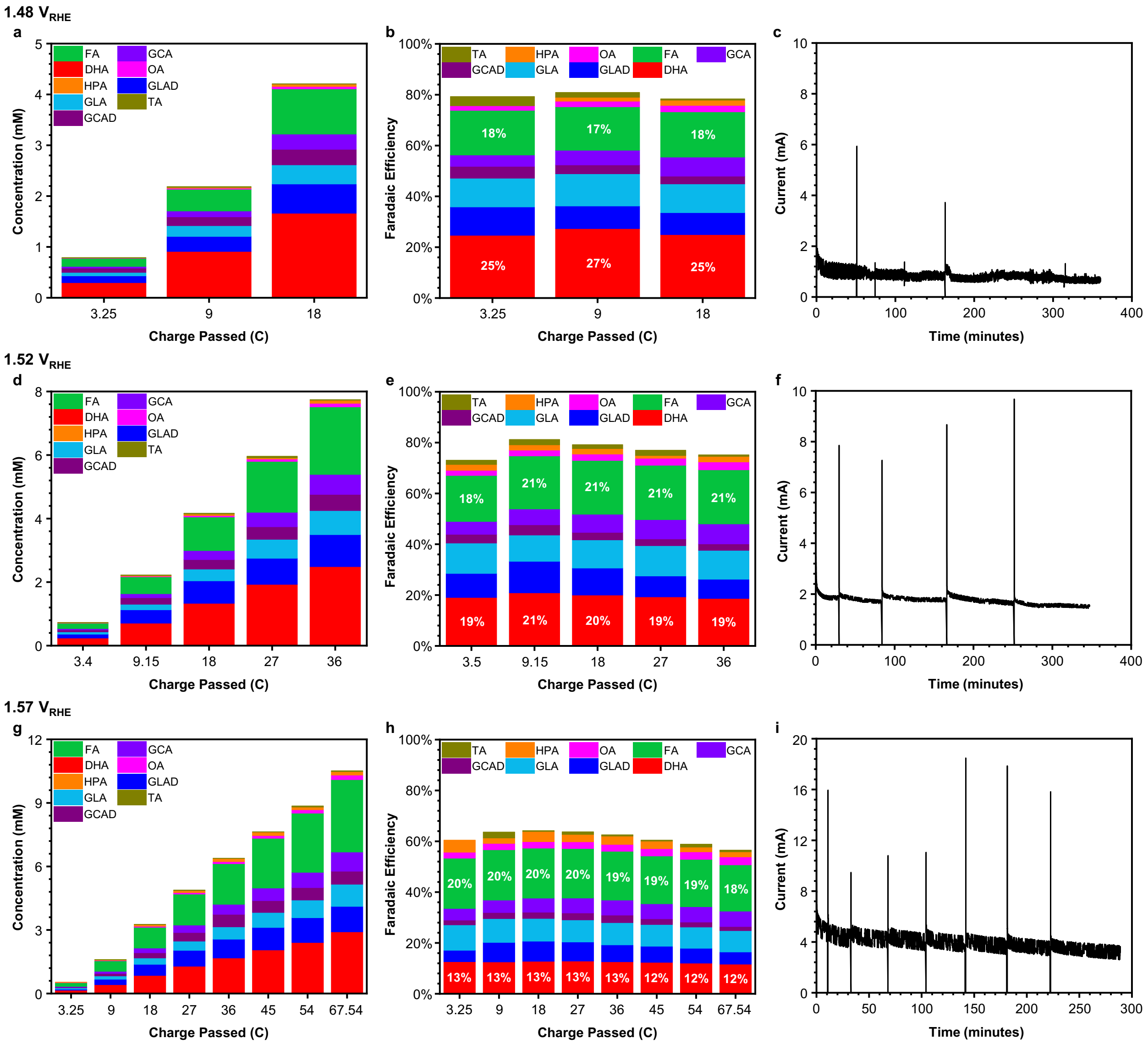

Supplementary Fig. 6. Glycerol oxidation results at pH 11 during electrolysis at a constant potential of 1.48 V_{RHE}, 1.52 V_{RHE}, or 1.57 V_{RHE}. **a, d, g** Product distributions showing product concentrations throughout electrolysis. **b, e, h** Faradaic efficiencies throughout electrolysis. The percentages in white indicate the specific FEs for DHA (red) and FA (green). **c, f, i** Current versus time profile for the electrolysis.

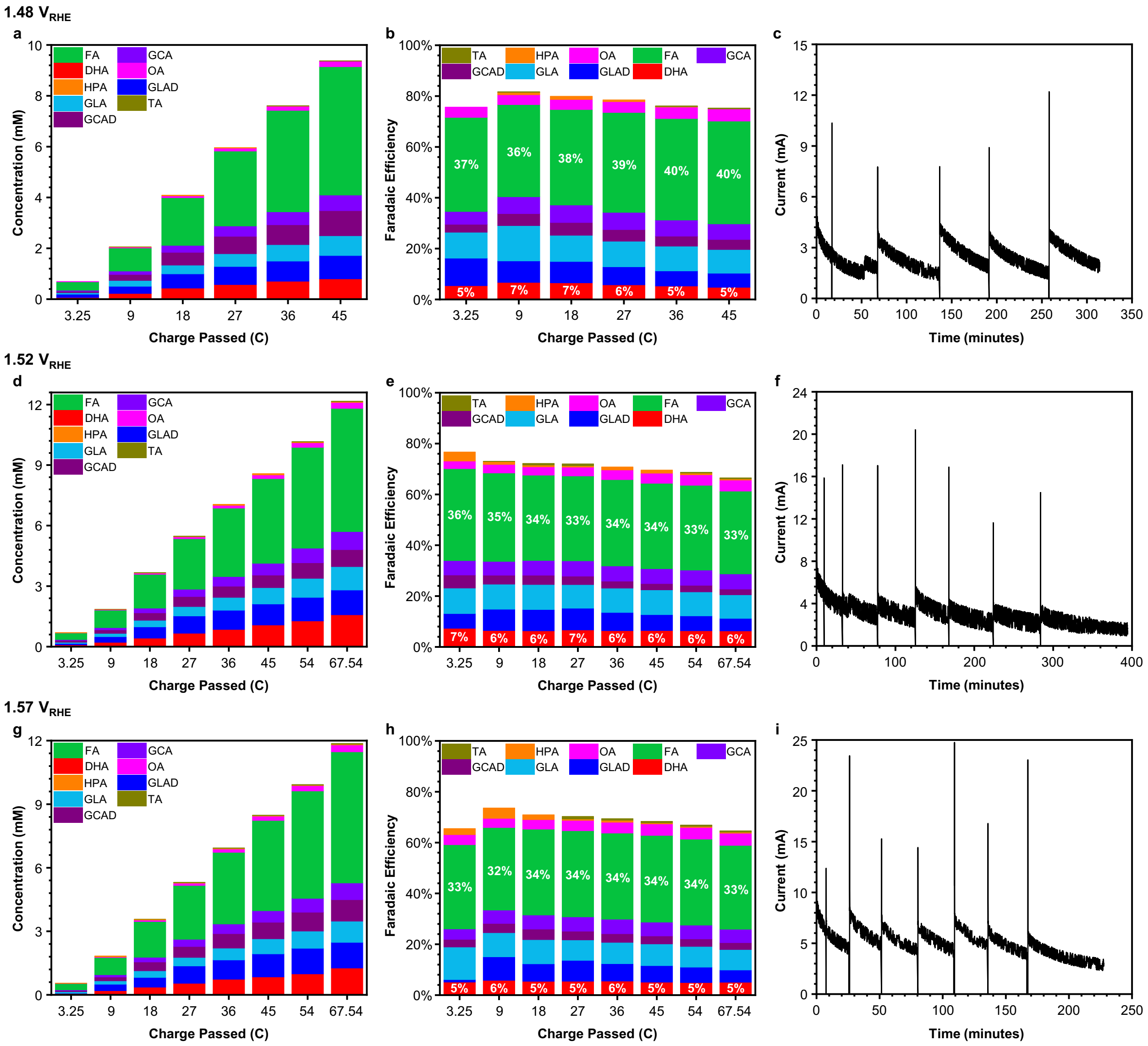

Supplementary Fig. 7. Glycerol oxidation results at pH 12 during electrolysis at a constant potential of 1.48 V_{RHE}, 1.52 V_{RHE}, or 1.57 V_{RHE}. **a, d, g** Product distributions showing product concentrations throughout electrolysis. **b, e, h** Faradaic efficiencies throughout electrolysis. The percentages in white indicate the specific FEs for DHA (red) and FA (green). **c, f, i** Current versus time profile for the electrolysis.

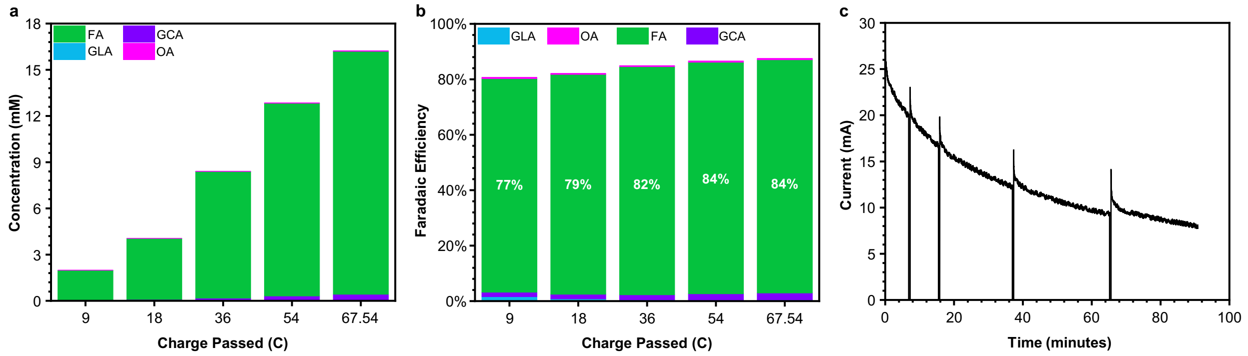

Supplementary Fig. 8. Glycerol oxidation results at pH 13 during electrolysis at a constant potential of 1.52 V_{RHE}. **a** Product distributions showing product concentrations throughout electrolysis. **b** Faradaic efficiencies throughout electrolysis. The percentages in white indicate the specific FE for FA (green). **c** Current versus time profile for the electrolysis.

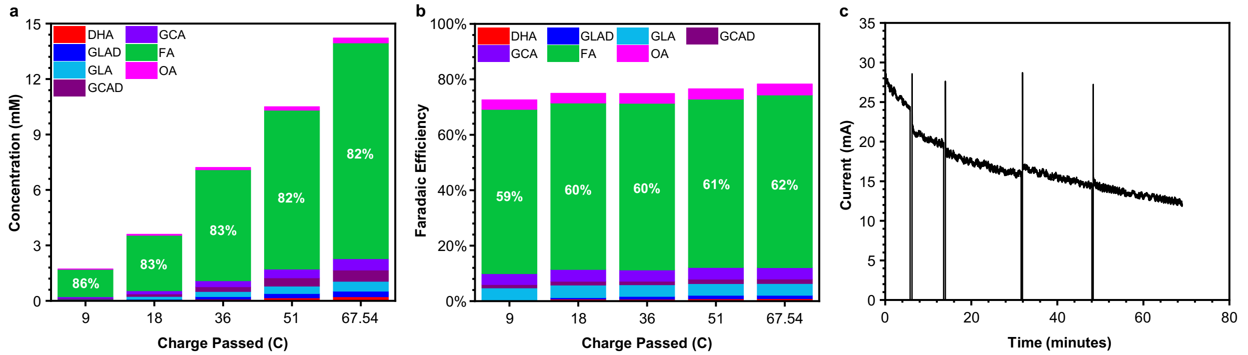

Supplementary Fig. 9. Glycerol oxidation results at pH 13 (solution prepared by adding KOH(s) to a 0.75 M H_3BO_3 solution to examine the effects of borate on the results) during electrolysis at a constant potential of 1.52 V_{RHE}. **a** Product distributions showing product concentrations throughout electrolysis. The percentages in white indicate the relative selectivity for FA (green). **b** Faradaic efficiencies throughout electrolysis. The percentages in white indicate the specific FE for FA (green). **c** Current versus time profile for the electrolysis.

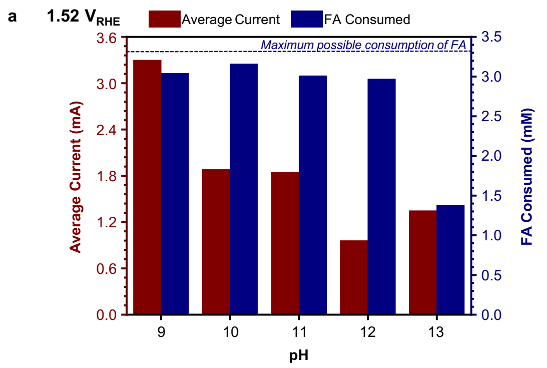

Supplementary Fig. 10. Oxidation results after constant potential electrolysis of FA. **a** FA oxidation results after electrolysis at a constant potential of 1.52 V_{RHE} under various pH conditions. **b** FA oxidation results after electrolysis at pH 10 under various potential conditions. The constant potentials were held until 9 C of charge had passed. The average current was calculated by dividing 9 C by the time it took to complete the electrolysis. During these experiments, FA is consumed but no other products are detected, indicating that FA is oxidized to CO_2 (that is trapped as HCO_3^- /CO_3^{2-} in our basic solutions). A maximum amount of 3.33 mM FA could be consumed by the 9 C passed if these reactions had 100% FE and selectivity towards CO_2 formation (a 2 e^- oxidation).

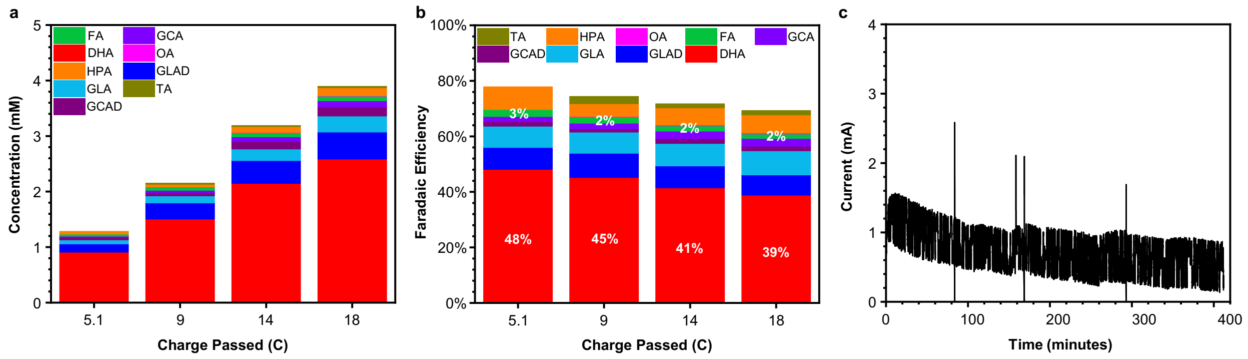

Supplementary Fig. 11. Glycerol oxidation results at pH 8 during electrolysis at a constant potential of 1.52 V_{RHE}. **a** Product distributions showing product concentrations throughout electrolysis. **b** Faradaic efficiencies throughout electrolysis. The percentages in white indicate the specific FEs for DHA (red) and FA (green). **c** Current versus time profile for the electrolysis.

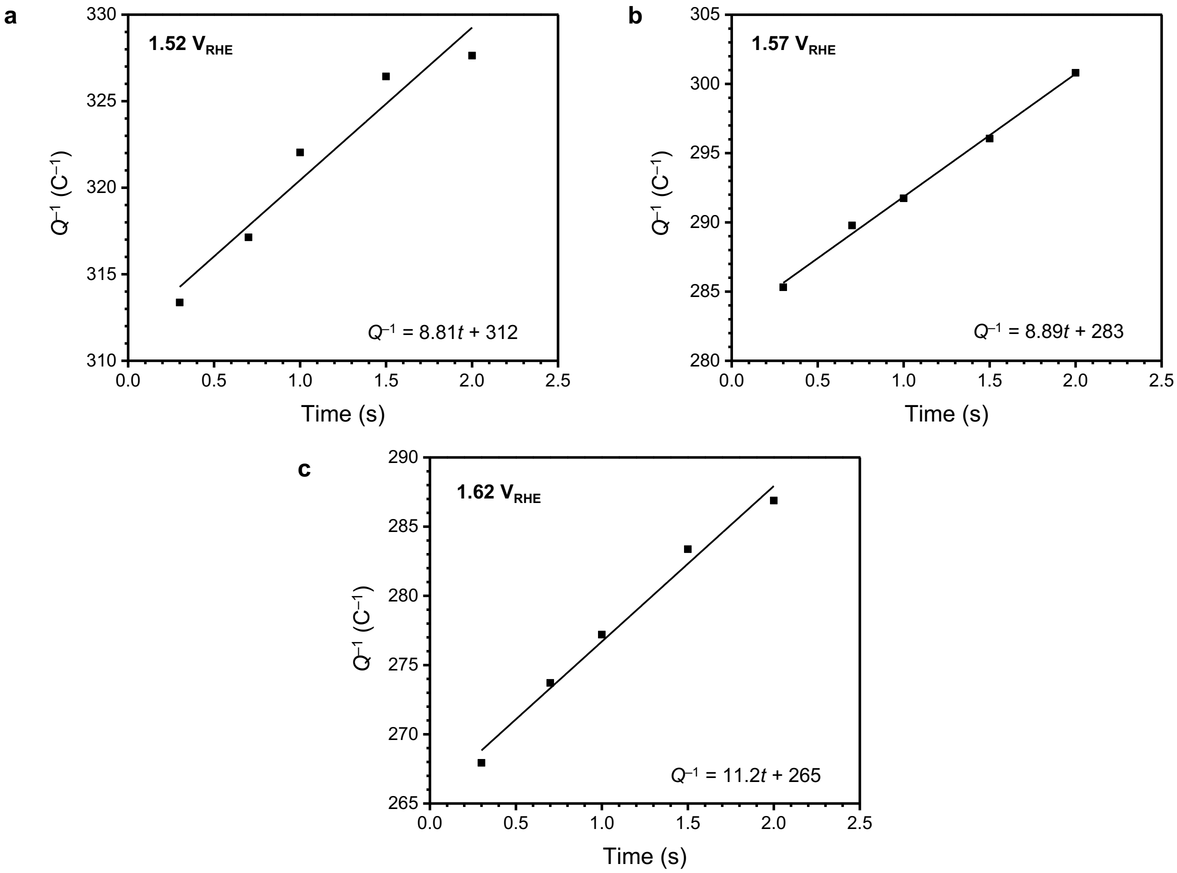

Supplementary Fig. 12. Q^{-1} vs t plots for rate deconvolution experiments carried out in a 25 mM glycerol, pH 9 solution. The potential applied in step 1 of the rate deconvolution procedure was **a** 1.52 V_{RHE}, **b** 1.57 V_{RHE}, **c** 1.62 V_{RHE}.

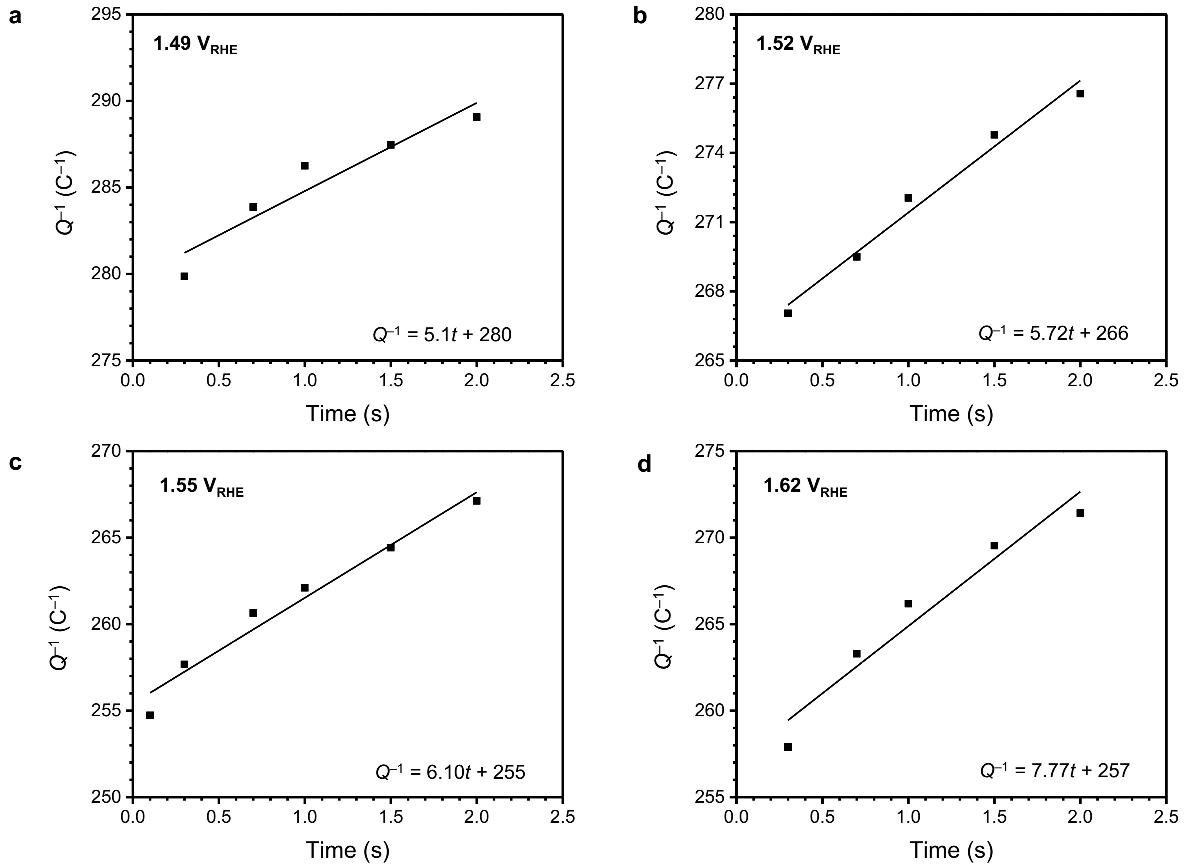

Supplementary Fig. 13. Q^{-1} vs t plots for rate deconvolution experiments carried out in a 25 mM glycerol, pH 10 solution. The potential applied in step 1 of the rate deconvolution procedure was **a** 1.49 V_{RHE}, **b** 1.52 V_{RHE}, **c** 1.55 V_{RHE}, **d** 1.62 V_{RHE}.

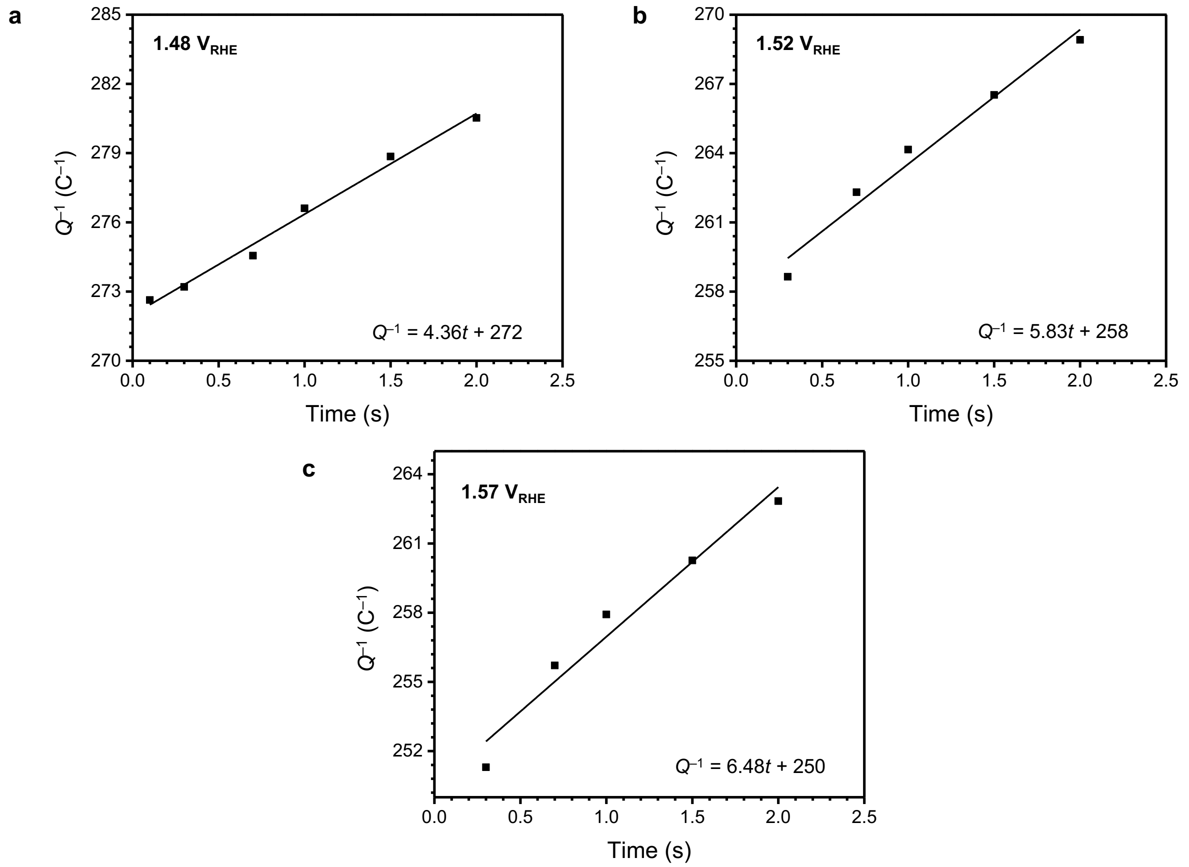

Supplementary Fig. 14. Q^{-1} vs t plots for rate deconvolution experiments carried out in a 25 mM glycerol, pH 11 solution. The potential applied in step 1 of the rate deconvolution procedure was **a** 1.48 V_{RHE}, **b** 1.52 V_{RHE}, **c** 1.57 V_{RHE}.

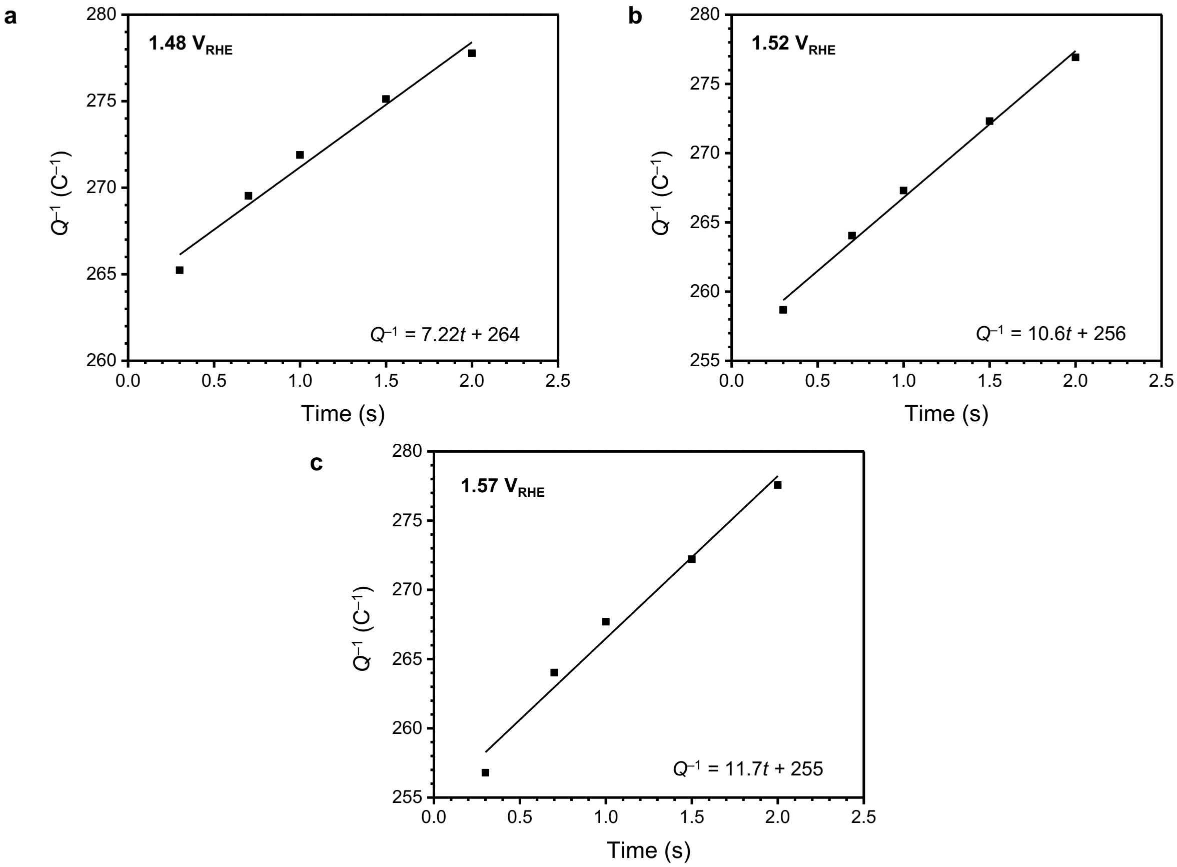

Supplementary Fig. 15. Q^{-1} vs t plots for rate deconvolution experiments carried out in a 25 mM glycerol, pH 12 solution. The potential applied in step 1 of the rate deconvolution procedure was **a** 1.48 V_{RHE}, **b** 1.52 V_{RHE}, **c** 1.57 V_{RHE}.
